# ARCN1 suppresses innate immune responses against respiratory syncytial virus by promoting STUB1-mediated IKKε degradation

**DOI:** 10.1371/journal.ppat.1013751

**Published:** 2025-12-04

**Authors:** Jiamin Cai, Yunfei Ye, Zhengrong Chen, Fei Xu, Xiaoping Li, Mengyun Wu, Ji Zhou, Yu Shao, Peijie Zhu, Jing Zhao, Jingjing Hu, Yufeng Wang, Cancheng Li, Xiaoyu Tian, Beibei Huang, Tian Xia, Wenjun Wang, Chuangli Hao, Yi Yang, Jinping Zhang

**Affiliations:** 1 The Fourth Affiliated Hospital, Institutes of Biology and Medical Sciences, Suzhou Medical College, Soochow University, Suzhou, People’s Republic of China; 2 Department of Respiratory Medicine, Children’s Hospital of Soochow University, Suzhou, China; 3 Department of Clinical Laboratory, Soochow University Affiliated No 1 People’s Hospital: First Affiliated Hospital of Soochow University, Suzhou, China; HVRI: Chinese Academy of Agricultural Sciences Harbin Veterinary Research Institute, CHINA

## Abstract

Respiratory syncytial virus (RSV), a filamentous, enveloped, negative-sense, single-stranded RNA virus, is a common cause of respiratory infections in pediatric and elderly populations, often leading to severe inflammation and lung damage. To date, there is no effective treatment for RSV infection. Therefore, elucidating the antiviral mechanism in RSV infection is critical for identifying potential effective therapeutic targets. Type I interferons (IFN-Is), especially IFN-β, play a key role in the immediate immune response against viral infection by inhibiting viral load and spread. Current studies have shown that ARCN1 (archain 1), the δ subunit of the coat protein complex I complex, is involved in intra-Golgi trafficking and protein transport from the Golgi apparatus back to the endoplasmic reticulum. In this study, we demonstrate that ARCN1 suppresses the anti-RSV innate immune response by promoting E3 ubiquitin ligase STUB1-mediated K48-linked polyubiquitination and subsequent proteasomal degradation of IKKε. Mechanistically, ARCN1 recognizes and binds to the δL motif of STUB1 via its flexible MHD domain while interacting with IKKε. This finding provides a theoretical basis for the development of new antiviral treatment strategies targeting ARCN1.

## Author summary

Intracellular trafficking between organelles is essential for maintaining key cellular functions and plays a crucial role in host antiviral immunity. The COPI complex, a central player in intracellular trafficking, may influence immune responses; however, it remains unclear whether specific COPI subunits regulate RNA sensor-mediated type I interferon (IFN-I) production during RSV infection. Clarifying this relationship could reveal new mechanisms of anti-RSV regulation and identify potential therapeutic targets. In this study, we demonstrate that RSV-induced upregulation of ARCN1, the δ subunit of the coat protein complex I (COPI), relies on IFN-β production and signaling. Based on this observation, we further demonstrate that ARCN1 acts as a negative regulator of IFN-β production during RSV infection. Mechanistically, ARCN1 enhances STUB1-mediated K48-linked polyubiquitination of IKKε, promoting its proteasomal degradation, inhibiting downstream antiviral responses, and ultimately impairing the host’s innate immunity to RSV.

## Introduction

Respiratory syncytial virus (RSV) is a prevalent human pathogen that causes respiratory tract infections in pediatric and elderly populations. It can cause severe complications, including pneumonia and bronchitis, and is distributed globally [1,2]. Despite decades of research, therapeutic options for RSV infection remain largely supportive, and no universally effective antiviral treatments are available. In recent years, there has been renewed focus on preventive strategies, especially vaccine development. Advances in structural biology have led to the stabilization of prefusion F protein antigens, facilitating the development of novel vaccine platforms such as mRNA-based and adjuvanted protein subunit formulations. These candidates have shown favorable immunogenicity and safety profiles in clinical trials, particularly among older adults [3,4]. However, current vaccines do not provide complete or long-lasting protection across all vulnerable populations, and the correlates of protective immunity remain poorly understood. Therefore, a deeper understanding of host antiviral immune mechanisms and viral factors of RSV pathogenesis is essential for guiding the development of next-generation vaccines and therapeutics.

Interferons (IFNs) are cytokines that play an important role in protection against viral defense, primarily through type I interferons (IFN-Is) [5]. Upon infection, RSV RNA, similar to that of other RNA viruses, activates toll-like receptor (TLR) and retinoic acid–inducible gene I (RIG-I) pathways to induce IFN-I production [6–8]. These pattern recognition receptors (PRR) activate a signaling cascade that promotes phosphorylation at Ser172 (S172) and subsequent activation of IKKε. Activated IKKε initiates the IFN-I signaling pathway by phosphorylating key transcription factors, including IRF3, IRF7, and STAT1, thereby eliciting antiviral responses and inhibiting virus replication [6,9]. During the innate immune response, IKKε phosphorylates specific proteins to counteract negative regulators of innate immune gene expression. For example, IKKε phosphorylates FAS-associated factor 1 (FAF1) at Ser556 (S556), relieving FAF1-mediated inhibition of mitochondrial antiviral-signaling protein (MAVS), a critical RNA-sensing adaptor [10].

Clinical reports have described the use of IFN-α1b for treating RSV infection by nebulized inhalation or intramuscular injection [11,12]. Although IFN-α1b has shown some therapeutic benefit for RSV infection, several challenges remain. The treatment must be initiated early, the treatment window is narrow, and clinical efficacy is inconsistent. With increasing understanding of the role of IFN-Is in RSV infection, researchers have begun investigating the relationship between regulatory molecules of IFN-I production and RSV to identify potential therapeutic targets, such as IFITM1 [13], SOCS1, and SOCS3 [14], etc. Although studies on IFN-I regulatory molecules in RSV infection are still ongoing, these discoveries may open new avenues for anti-RSV therapy.

Archain 1 (ARCN1) is the δ subunit of coat protein complex I (COPI), which also includes six other subunit proteins: α-COP (COPA), β-COP (COPB1), β’-COP (COPB2), ε-COP (COPE), γ-COP (COPG), and ζ-COP (COPZ1). COPI mediates intra- Golgi apparatus (Golgi) protein transport and retrograde trafficking from the Golgi to the endoplasmic reticulum (ER), processes essential for the function of almost all cell types [15–17]. The COPI complex supports the life cycle of multiple negative-strand RNA viruses, including paramyxoviruses, by facilitating critical steps in viral replication and assembly [18–20].

This proviral role of the COPI complex has also been predicted by Anderson et al. [21] using meta-analyses across different viruses. A specific subunit of the COPI complex, COPA, has been shown to play an important role in innate immunity, especially in antiviral immunity. Watkin et al. [22] demonstrated that autosomal dominant mutations in COPA (encoding the α-COP) cause COPA syndrome, characterized by dysregulation of innate and adaptive immunity. Furthermore, Volpi et al. [23] demonstrated elevated transcription of IFN-Is and interferon-stimulated genes (ISGs) in the peripheral blood of patients with COPA syndrome. Further studies showed that COPA deficiency enhances IFN-I production via impaired STING retrieval from the Golgi to the ER, leading to aberrant activation of the cGAS–STING pathway [24]. However, it remains unclear whether specific COPI subunits regulate RNA sensor–mediated IFN-I production during RSV infection. Our initial experimental data showed that COPD (also known as ARCN1) is significantly upregulated during RSV infection, suggesting its potential involvement in host antiviral responses and the significance of further exploring the underlying mechanism.

Further mechanistic analyses revealed that ARCN1 facilitates the interaction between STUB1 and IKKε. STUB1 subsequently catalyzes K48-linked polyubiquitination of IKKε at Lys231 (a key kinase in the RIG-I signaling pathway), targeting it for proteasomal degradation and thereby attenuating IFN-β signaling. Consistent with this mechanism, upregulation of ARCN1 during RSV infection diminishes IFN-β production through STUB1-dependent degradation of IKKε. Notably, prior studies have shown that, in the context of RSV infection, type I interferons (IFN-I) not only induce antiviral programs but also robustly amplify pulmonary proinflammatory cytokine production; excessive or sustained IFN-I signaling exacerbates lung inflammation and tissue damage, worsening disease progression [25,26]. In this context, increased ARCN1 expression likely functions as a host-protective feedback mechanism that restrains hyperactivation of the IFN-I pathway and limits immunopathology. Together, these findings establish ARCN1 as a negative regulator of antiviral signaling that constrains IFN-β responses while helping to preserve immune homeostasis during RSV infection, providing new insight into the host response to RSV.

## Results

### RSV infection leads to increased ARCN1 expression in macrophages

Intracellular transport between organelles is essential for fundamental biological processes. During viral infection, trafficking machinery can be used by viruses to facilitate infection [27–29]. However, research on host trafficking protein–virus interactions remain limited, hindering antiviral therapeutic development. To investigate the role of trafficking proteins in RSV infection, we established an infection model using respiratory syncytial virus strain L19 tagged with mCherry (RSV-L19-mCherry; multiplicity of infection (MOI) =10) in RAW264.7 cells. Representative fluorescence images confirmed successful infection, as evidenced by distinct red fluorescence in these cells 24 h postinfection (S1a Fig). Protein mass spectrometry was performed to investigate the impact of RSV infection on host macrophage proteins expression. Hierarchical clustering of differentially expressed trafficking proteins revealed clear separation between RSV-L19-infected and control groups. Notably, the vesicle trafficking protein ARCN1 exhibited stable and significant upregulation following infection (S2a, S2b Fig), suggesting a previously unrecognized role in RSV pathogenesis. Therefore, ARCN1 was selected as the target for further investigation.

**Fig 1:**
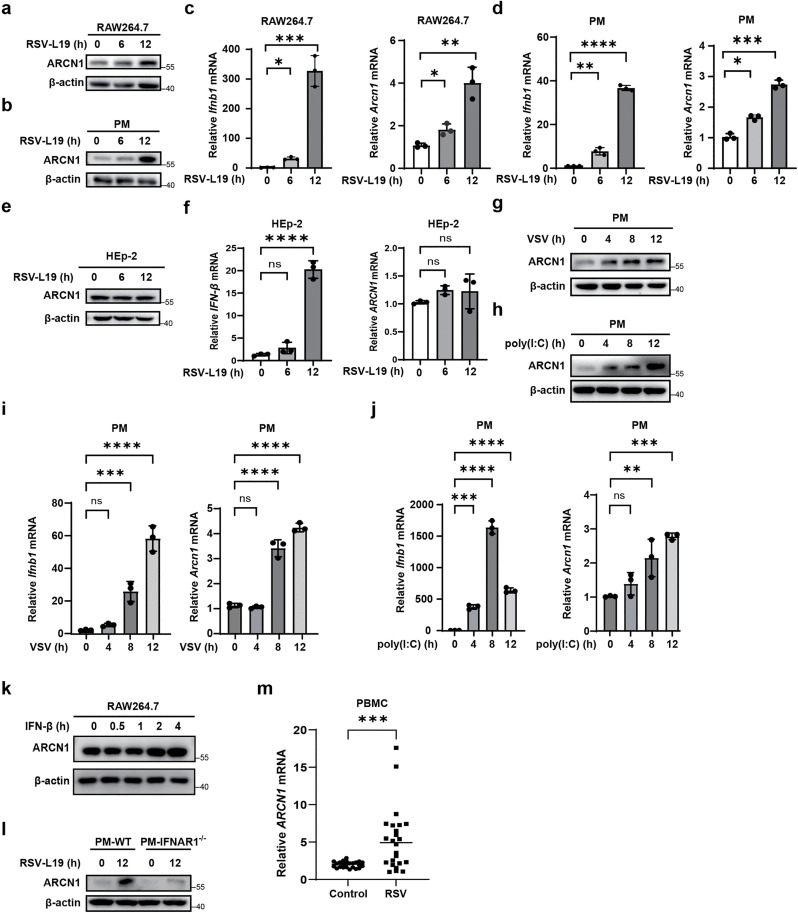
RSV infection leads to increased ARCN1 expression in macrophages. **(a–b)** The protein expression of ARCN1 was detected by Western blot after RAW264.7 **(a)** and PMs **(b)** were infected with RSV-L19 (MOI = 10) at different time points. n = 3 biological replicates. **(c–d)** The mRNA expressions of *Ifnb1* and *Arcn1* were detected by RT-qPCR after RAW264.7 **(c)** and PMs **(d)** were infected with RSV-L19 (MOI = 10) at different time points. The data are presented as the means ±SEM. n = 3 biological replicates. The expressions were normalized to 18S mRNA and further normalized to the 0-h time point. **(e)** The protein expression of ARCN1 was detected by Western blot after HEp-2 cells were infected with RSV-L19 (MOI = 10) at different time points. n = 3 biological replicates. **(f)** The mRNA expressions of *IFN-β* and *ARCN1* were detected by RT-qPCR after HEp-2 cells were infected with RSV-L19 (MOI = 10) at different time points. The data are presented as the means ±SEM. n = 3 biological replicates. The expressions were normalized to 18S mRNA and further normalized to the 0-h time point. **(g–h)** The protein expression of ARCN1 was detected by Western blot after PMs were treated with VSV (MOI = 0.1) **(g)** or poly(I:C) (1 μg/mL) (h) at different time points. n = 3 biological replicates. **(i–j)** RT-qPCR was used to detect the mRNA expressions of *Ifnb1* and *Arcn1* after PMs were treated with VSV (MOI = 0.1) **(i)** or poly(I:C) (1 μg/mL) **(j)** at different time points. Data are presented as the mean ±SEM. n = 3 biological replicates. The expressions were normalized to 18S mRNA and further normalized to the 0-h time point. **(k)** Western blot was used to detect the protein expression of ARCN1 after RAW264.7 cells were stimulated with IFN-β (3,000 U/mL) at different time points. n = 3 biological replicates. **(l)** Western blot of ARCN1 in peritoneal macrophages (PMs) of WT and IFNAR1^−/−^ mice infected with RSV-L19 (MOI = 10). n = 3 biological replicates. **(m)** mRNA was extracted from PBMCs of the control group (n = 24) and RSV-infected children (n = 24), followed by RT-qPCR detection of the mRNA expression of *ARCN1* (each data point represents one person). The *P*-value was determined using an unpaired t-test. ns, not significant (*P* > 0.05); *P < 0.05; **P < 0.01; ***P < 0.001; ****P < 0.0001.

To validate these proteomic findings, ARCN1 expression was assessed via Western blot and quantitative real-time polymerase chain reaction (RT-qPCR) in RAW264.7 cells and mouse primary peritoneal macrophages (PMs) infected with RSV-L19 (MOI = 10). Phenotypic fluorescence images confirmed successful infection, as evidenced by clear mCherry red fluorescence in both cell types at 12 h postinfection (S1a Fig). The results confirmed that RSV-L19 infection significantly upregulated ARCN1 protein and mRNA levels in both macrophage populations (Fig 1a–1d). Because RSV-L19 is a laboratory-adapted strain, we next included RSV-A2—a widely used wild-type RSV strain with biological properties distinct from RSV-L19 [30–34]—to determine whether ARCN1 induction is genuinely driven by RSV infection rather than a strain-specific artifact. To contextualize strain differences, we also compiled, based on published literature, a comparative summary of the biological properties of RSV-L19 versus RSV-A2, covering replication kinetics, thermal/structural stability, cytopathology, immune bias, type I IFN response, in vivo viral load, airway hyperreactivity, pathogenicity, and experimental use (S1 Table). To equalize viral input, we performed parallel infections of RAW264.7 cells normalized by PFU, using MOIs of 10 (RSV-L19) and 0.1 (RSV-A2). At 12 h postinfection, RSV M2-1 protein was quantified by Western blot, and *RSV-F* and *Ifnb1* mRNAs were measured by RT-qPCR. Under PFU-normalized conditions, RSV-A2 displayed higher infectivity and elicited stronger *Ifnb1* induction than RSV-L19 (S3a–S3b Fig). We then infected RAW264.7 cells and PMs with RSV-A2 (MOI = 0.1) and assessed ARCN1 expression by Western blot and RT-qPCR. RSV-A2 infection robustly upregulated ARCN1 at both the protein and mRNA levels in both cell types (S3c–S3f Fig), indicating that ARCN1 induction is a conserved macrophage response to RSV and corroborating that the increase is a bona fide RSV-induced effect rather than a property unique to the L19 strain. Given that macrophages are the primary producers of IFN-Is and that airway epithelial cells are the primary target cells for RSV infection, we next investigated whether ARCN1 is also involved in the antiviral response of epithelial cells to RSV. Therefore, we selected HEp-2, an epithelial cell line commonly used in RSV research [35,36], and infected it with RSV-L19 (MOI = 10) to examine the expression of ARCN1. However, ARCN1 expression remained largely unchanged in HEp-2 cells after RSV-L19 infection, suggesting that RSV-induced ARCN1 upregulation is cell type-specific (Fig 1e, 1f). These results consistently demonstrate that RSV infection induces ARCN1 expression at both protein and mRNA levels in macrophages.

To further confirm whether ARCN1 upregulation is RSV-specific, mouse primary PMs were stimulated with vesicular stomatitis virus (VSV, MOI = 0.1) or the viral double-stranded RNA mimic poly(I:C). RT-qPCR and Western blot analyses revealed that both VSV and poly(I:C) significantly increase ARCN1 protein and mRNA expression in PMs (Fig 1g–1j), suggesting that RSV-induced ARCN1 upregulation is not unique to RSV infection.

Notably, RSV-L19, RSV-A2, VSV, and poly(I:C) treatments significantly induced *Ifnb1* mRNA expression in macrophages. This observation prompted an investigation into whether ARCN1 upregulation is dependent on IFN-β signaling. Therefore, we stimulated RAW264.7 cells with recombinant mouse IFN-β (3000 U/mL), and Western blot analysis confirmed significant induction of ARCN1 protein expression (Fig 1k). Importantly, compared with wild-type (WT) mouse PMs, ARCN1 expression in IFN-I receptor–deficient (IFNAR1 ⁻ /⁻) mouse PMs was not significantly upregulated following RSV-L19 infection (Fig 1l). These results collectively indicate that RSV-induced ARCN1 upregulation depends on IFN-β production and signaling through IFNAR.

To validate these findings in a clinical context, *ARCN1* mRNA expression was measured in peripheral blood mononuclear cells (PBMCs) from RSV-infected children (n = 24) and control group children (n = 24) using RT-qPCR. The results showed that RSV infection significantly upregulated the mRNA expression of *ARCN1* in peripheral blood (Fig 1m). This finding confirms the universality of ARCN1 upregulation in RSV-infected children and suggests its potential value in clinical diagnosis and the study of RSV pathogenesis.

### ARCN1 facilitates the viral load of RNA viruses

**Fig 2:**
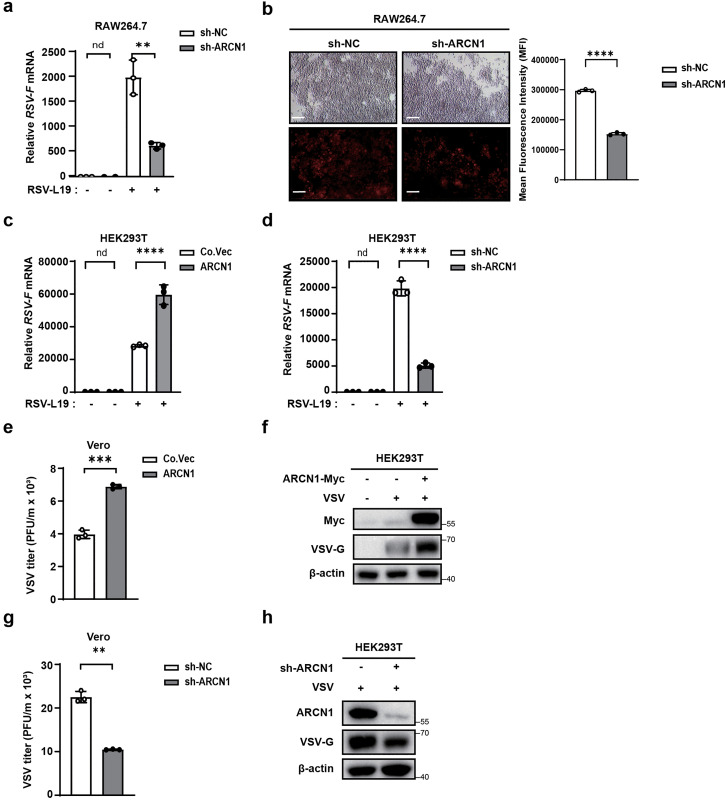
ARCN1 facilitates the viral load of RNA viruses. **(a)** The mRNA expression of *RSV-F* in sh-NC-RAW264.7 and sh-ARCN1-RAW264.7 infected with RSV-L19 (MOI = 10) for 12 h was detected by RT-qPCR. Data are presented as the mean ±SEM. n = 3 biological replicates. The expressions were normalized to 18S mRNA and further normalized to the sh-NC-0-h sample. **(b)** The fluorescence of RSV-L19 was detected by fluorescence microscopy after sh-NC-RAW264.7 and sh-ARCN1-RAW264.7 were infected with RSV-L19-mCherry (MOI = 10) for 24 h. Scale bar = 200 μm. ImageJ software was used to perform quantitative statistical analysis of the red fluorescence signal. n = 3 biological replicates. **(c)** HEK293T cells were transfected with control vector (Co.Vec) or ARCN1-Myc (ARCN1) plasmids for 36 h and then infected with RSV-L19 (MOI = 10) for 12 h. The mRNA expression of *RSV-F* in the cells was detected by RT-qPCR. Data are presented as the means ±SEM. n = 3 biological replicates. The expressions were normalized to 18S mRNA and further normalized to the Co.Vec-0-h sample. **(d)** HEK293T cells were transfected with lentivirus encoding pLL3.7 vector (sh-NC) or shRNA plasmid specific for ARCN1 (sh-ARCN1) for 36 h and then infected with RSV-L19 (MOI = 10) for 12 h. The mRNA expression of *RSV-F* in the cells was detected by RT-qPCR. Data are presented as the mean ±SEM. n = 3 biological replicates. The expressions were normalized to 18S mRNA and further normalized to the sh-NC-0-h sample. **(e–f)** HEK293T cells were transfected with Co.Vec or ARCN1-Myc for 36 h and then infected with VSV (MOI = 0.1) for 12 h. The VSV viral load in the culture supernatant of HEK293T cells was detected by plaque assay in Vero cells **(e)**. The protein expressions of VSV-G and exogenous ARCN1 in HEK293T cells were then detected by Western blot **(f)**. n = 3 biological replicates. **(g–h)** HEK293T cells were transfected with sh-NC or sh-ARCN1 for 36 h and then infected with VSV (MOI = 0.1) for 12 h. The VSV viral load in the culture supernatant of HEK293T cells was detected by plaque assay in Vero cells **(g)**. The protein expressions of ARCN1 and VSV-G in HEK293T cells were then detected by Western blot **(h)**. n = 3 biological replicates. **(a, c–d)** The *P*-value was determined using a two-way ANOVA test. **(b, e, g)** The *P*-value was determined using an unpaired t-test. nd, not detected (below assay detection limit); **P < 0.01; ***P < 0.001; ****P < 0.0001.

To investigate whether ARCN1 regulates RSV replication, we first generated stable RAW264.7 cell lines with an ARCN1 knockdown line (sh-ARCN1-RAW264.7) and a control line (sh-NC-RAW264.7). Successful ARCN1 knockdown was confirmed via RT-qPCR and Western blot (S4a–S4b Fig). Cells were then infected with RSV-L19 (MOI = 10), and *RSV-F* mRNA levels were measured via RT-qPCR. ARCN1 knockdown significantly reduced *RSV-F* mRNA expression (Fig 2a). Immunofluorescence analysis of RSV-L19–mCherry infection further demonstrated a marked decrease in RSV-positive cells following ARCN1 knockdown, indicating reduced viral load. To provide a quantitative assessment of this phenotype, RSV-positive cells were quantified using ImageJ, and the corresponding statistical analyses were consistent with the immunofluorescence images, confirming that ARCN1 knockdown reduces the extent of RSV infection (Fig 2b).

To determine whether ARCN1’s effect was cell-type independent, ARCN1 expression was modulated in HEK293T cells via plasmid transfection. We first confirmed successful modulation by verifying ARCN1 protein and mRNA levels in these transfected HEK293T cells (S4c–S4f Fig). Results showed that ARCN1 overexpression significantly enhanced *RSV-F* expression (Fig 2c), whereas ARCN1 knockdown significantly inhibited it (Fig 2d), confirming that ARCN1 promotes RSV replication. To investigate whether this proviral effect was unique to RSV, we further examined its effect on the viral load of VSV (MOI = 0.1). Plaque assays revealed that ARCN1 overexpression significantly increased VSV titers (Fig 2e), and Western blot revealed that ARCN1 overexpression enhanced VSV-G protein expression in HEK293T cells (Fig 2f). Conversely, ARCN1 knockdown significantly reduced VSV titers (Fig 2g) and VSV-G protein levels (Fig 2h).

Collectively, these findings indicate that ARCN1 promotes the viral load of RSV and VSV, suggesting its broad proviral functions during RNA virus infection. This is consistent with previous reports demonstrating that ARCN1 is involved in the life cycle of multiple negative-strand RNA viruses [21].

### ARCN1 promotes the viral load of RNA viruses by negatively regulating IFN-β

**Fig 3:**
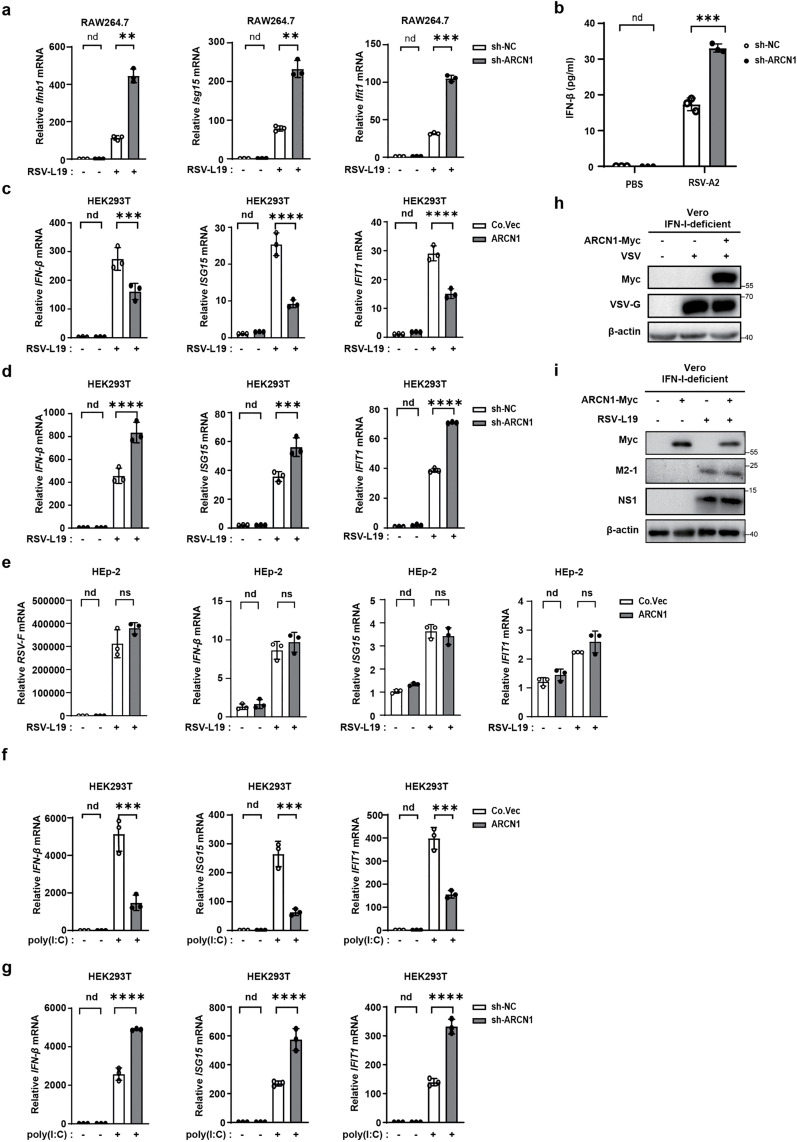
ARCN1 promotes the viral load of RNA viruses by negatively regulating IFN-β. **(a)** The mRNA expression of *Ifnb1*, *Isg15*, and *Ifit1* in sh-NC-RAW264.7 and sh-ARCN1-RAW264.7 infected with RSV-L19 (MOI = 10) for 12 h was detected by RT-qPCR. Data are presented as the mean ±SEM. n = 3 biological replicates. The expressions were normalized to 18S mRNA and further normalized to the sh-NC-0-h sample. **(b)** ELISA of the IFN-β expression in supernatant from sh-NC-RAW264.7 and sh-ARCN1-RAW264.7 cells infected with RSV-A2 (MOI = 0.1) for 24 h. n = 3 biological replicates. **(c)** HEK293T cells were transfected with control vector (Co.Vec) or ARCN1-Myc (ARCN1) plasmids for 36 h and then infected with RSV-L19 (MOI = 10) for 12 h. The mRNA expression of *IFN-β, ISG15*, and *IFIT1* in the cells was detected by RT-qPCR. Data are presented as the mean ±SEM. n = 3 biological replicates. The expressions were normalized to 18S mRNA and further normalized to the Co.Vec-0-h sample. **(d)** HEK293T cells were transfected with lentivirus encoding pLL3.7 vector (sh-NC) or shRNA plasmid specific for ARCN1 (sh-ARCN1) for 36 h and then infected with RSV-L19 (MOI = 10) for 12 h. The mRNA expression of *IFN-β*, *ISG15*, and *IFIT1* in the cells was detected by RT-qPCR. Data are presented as the mean ±SEM. n = 3 biological replicates. The expressions were normalized to 18S mRNA and further normalized to the sh-NC-0-h sample. **(e)** Hep-2 cells were transfected with control vector (Co.Vec) or ARCN1-Myc (ARCN1) plasmids for 36 h and then infected with RSV-L19 (MOI = 10) for 12 h. The mRNA expression of *RSV-F, IFN-β, ISG15*, and *IFIT1* in the cells was detected by RT-qPCR. Data are presented as the mean ±SEM. n = 3 biological replicates. The expressions were normalized to 18S mRNA and further normalized to the Co.Vec-0-h sample. **(f)** HEK293T cells were transfected with control vector (Co.Vec) or ARCN1-Myc (ARCN1) plasmids for 36 h and then treated with poly(I:C) (1 μg/mL) for 12 h. The mRNA expression of *IFN-β*, *ISG15*, and *IFIT1* in the cells was detected by RT-qPCR. Data are presented as the mean ±SEM. n = 3 biological replicates. The expressions were normalized to 18S mRNA and further normalized to the Co.Vec-0-h sample. **(g)** HEK293T cells were transfected with lentivirus encoding pLL3.7 vector (sh-NC) or shRNA plasmid specific for ARCN1 (sh-ARCN1) for 36 h and then treated with poly(I:C) (1 μg/mL) for 12 h. The mRNA expression of *IFN-β*, *ISG15*, and *IFIT1* in the cells was detected by RT-qPCR. Data are presented as the mean ±SEM. n = 3 biological replicates. The expressions were normalized to 18S mRNA and further normalized to the sh-NC-0h sample. **(h)** Vero cells were transfected with Co.Vec or ARCN1-Myc plasmids for 36 h and then infected with VSV (MOI = 0.1) for 12 h. The protein expressions of VSV-G and exogenous ARCN1 were then detected by Western blot. n = 3 biological replicates. **(i)** Vero cells were transfected with Co.Vec or ARCN1-Myc plasmids for 36 h and then infected with RSV-L19 (MOI = 10) for 12 h. The protein expressions of RSV-M2-1 and RSV-NS1 were then detected by Western blot. n = 3 biological replicates. (a–g) The *P*-value was determined using multiple unpaired t-tests. nd, not detected (below assay detection limit); ns, not significant (*P* > 0.05); **P < 0.01; ***P < 0.001; ****P < 0.0001.

To elucidate the mechanism by which ARCN1 promotes the viral load of RNA viruses, we investigated its role in regulating IFN-β expression. Following RSV-L19 infection (MOI = 10) of sh-ARCN1-RAW264.7 cells for 12 h, RT-qPCR analysis revealed significantly increased mRNA levels of *Ifnb1* and interferon-stimulated genes *Isg15* and *Ifit1* compared to sh-NC-RAW264.7 controls (Fig 3a), suggesting that ARCN1 suppresses RSV-induced IFN-β signaling. ELISA confirmed that ARCN1 knockdown significantly increased IFN-β secretion in the culture supernatant of RSV-A2-infected (MOI = 0.1) sh-ARCN1-RAW264.7 cells (Fig 3b).

To assess whether this regulation is cell-type independent, we modulated ARCN1 expression in HEK293T cells before RSV-L19 infection (MOI = 10). ARCN1 overexpression significantly inhibited RSV-induced mRNA expression of *IFN-β*, *ISG15*, and *IFIT1* (Fig 3c), whereas ARCN1 knockdown significantly enhanced their expression (Fig 3d). Because RSV primarily infects airway epithelial cells, we selected the HEp-2 epithelial cell line and overexpressed ARCN1 to assess its role in epithelial antiviral responses (S4g–S4h Fig). Following RSV-L19 infection (MOI = 10), ARCN1 overexpression did not alter *RSV-F* transcript levels, indicating no discernible impact on epithelial viral burden (Fig 3e). Consistently, ARCN1 overexpression exerted minimal effects on *IFN-β*, *ISG15*, and *IFIT1* mRNA expression as determined by RT-qPCR (Fig 3e). Based on the data from mentioned above, the expression level of ARCN1 remains almost unchanged after HEp-2 cells infection with RSV-L19 (Fig 1e, 1f). Taken together, these findings indicate that ARCN1 has limited regulatory activity in epithelial contexts and likely functions in a cell type–specific manner. A plausible explanation is the relatively attenuated IFN-β signaling in HEp-2 cells, which may constrain ARCN1-dependent modulation of the IKKε–IRF3–STAT1/2 axis. Accordingly, ARCN1-based interventions may be more effective in macrophage settings and could be prioritized or combined with macrophage-targeted approaches in therapeutic strategies for RSV.

To determine whether ARCN1 broadly suppresses antiviral responses triggered by viral RNA, we stimulated cells with poly(I:C) to activate the IFN-I pathway. ARCN1 overexpression markedly suppressed poly(I:C)-induced *IFN-β*, *ISG15*, and *IFIT1* expression (Fig 3f), whereas ARCN1 knockdown significantly enhanced their expression (Fig 3g). Collectively, these data demonstrate that ARCN1 inhibits host antiviral responses by negatively regulating IFN-β production.

Furthermore, to establish whether ARCN1’s proviral activity depends on the IFN-I pathway, we utilized IFN-I-deficient Vero cells. ARCN1 overexpression did not enhance VSV-G protein expression during VSV infection (MOI = 0.1) (Fig 3h) or RSV M2-1 and NS1 protein levels during RSV-L19 infection (MOI = 10) (Fig 3i). The absence of ARCN1’s proviral effect in IFN-I-deficient cells demonstrates that its promotion of RNA virus replication is dependent on IFN-I signaling. Thus, ARCN1 facilitates the viral load of RNA viruses by suppressing IFN-β signaling.

### ARCN1 inhibits IFN-β production by binding to IKKε

**Fig 4:**
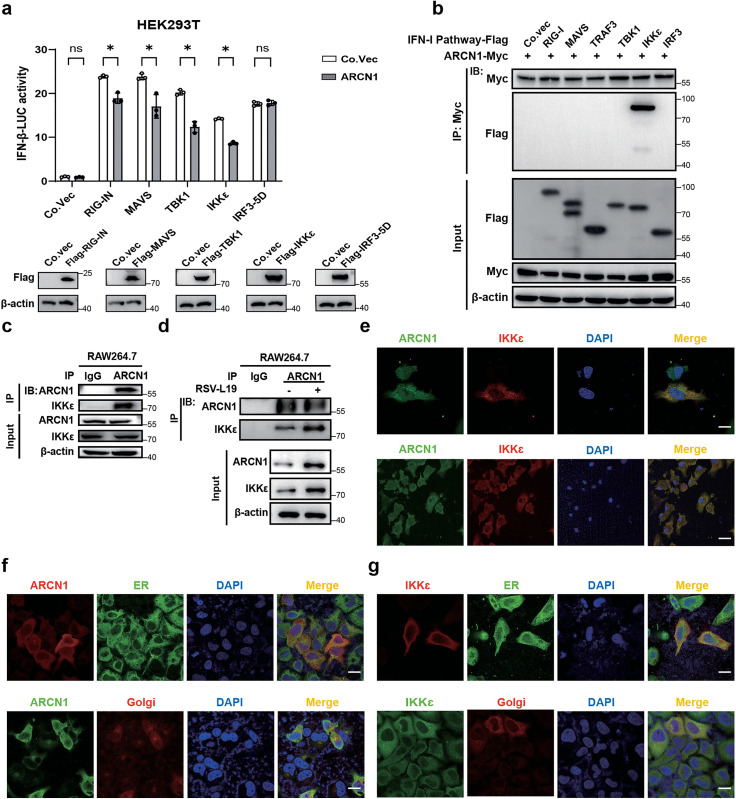
ARCN1 inhibits IFN-β production by binding to IKKε. **(a)** HEK293T cells were co-transfected with the control vector (Co.Vec) or RIG-I-N/MAVS/TBK1/IKKε/IRF3-5D plasmids, together with ARCN1-Myc, IFN-β promoter reporter plasmid, and Renilla plasmid for 36 h. Then, the IFN-β luciferase activity was analysed by luciferase reporter assay. Western blot of RIG-IN-Flag, MAVS-Flag, TBK1-Flag, IKKε-Flag, and IRF3-5D-Flag plasmids in HEK293T cells was performed using an anti-Flag antibody. n = 3 biological replicates. **(b)** Co-IP analysis of ARCN1-Myc along with the control vector-Flag (Co.Vec), RIG-I-Flag, MAVS-Flag, TRAF3-Flag, TBK1-Flag, IKKε-Flag, and IRF3-Flag plasmids in the HEK293T cells was performed with an anti-Myc antibody. n = 3 biological replicates. **(c)** Co-IP analysis of the interaction between endogenous ARCN1 and IKKε in RAW264.7 was performed using an anti-ARCN1 antibody. n = 3 biological replicates. **(d)** Co-IP analysis of the interaction between ARCN1 and IKKε was performed using an anti-ARCN1 antibody after RAW264.7 cells were infected with RSV-L19 (MOI = 10) for 12 h. n = 3 biological replicates. **(e)** HeLa cells were co-transfected with ARCN1-Myc and IKKε-Flag plasmids, immunofluorescently stained with Myc (green) and Flag (red) antibodies, and the co-localization was detected by laser confocal microscopy. Scale bar = 10 μm. Immunostaining of mouse peritoneal macrophages (PMs) stained with ARCN1 (green) and IKKε (red) antibodies through laser confocal microscopy. Scale bar = 10 μm. **(f)** HeLa cells were infected with RSV-A2 (MOI = 0.1) for 12 h; immunostaining of HeLa cells stained with ARCN1 (green/ red), BAP31 (green), and Giantin (red) antibodies through laser confocal microscopy. Scale bar = 10 μm. **(g)** HeLa cells were infected with RSV-A2 (MOI = 0.1) for 12 h; immunostaining of HeLa cells stained with IKKε (green/ red), BAP31 (green), and Giantin (red) antibodies through laser confocal microscopy. Scale bar = 10 μm. **(a)** The *P*-value was determined using multiple unpaired t-tests. ns, not significant (*P* > 0.05); *P < 0.05.

RSV RNA, like RNA from other RNA viruses, activates TLR and RIG-I pathways upon infection to induce IFN-Is production [37]. To delineate the mechanism underlying ARCN1-mediated IFN-β suppression, we first assessed its impact on key RIG-I pathway molecules using an IFN-β promoter–driven luciferase reporter assay in HEK293T cells. ARCN1 overexpression significantly inhibited IFN-β promoter activation induced by upstream signaling molecules (RIG-I, MAVS, TBK1, and IKKε) but had no significant effect on promoter activation by IRF3-5D (Fig 4a). This suggested IKKε as the target of ARCN1 within the IFN-I pathway. To identify ARCN1’s specific binding molecule, we performed co-immunoprecipitation (Co-IP) assays with key IFN-I pathway components. ARCN1 specifically interacted with IKKε, but not with RIG-I, MAVS, TBK1, TRAF3, or IRF3 (Fig 4b). This endogenous interaction was confirmed in RAW264.7 cells (Fig 4c) and was significantly enhanced 12 h after RSV-L19 infection (MOI = 10) (Fig 4d), suggesting that ARCN1-IKKε interaction is more active during RSV infection.

Consistent with prior observations, confocal microscopy of HeLa cells ectopically coexpressing ARCN1 and IKKε revealed robust colocalization (Fig 4e), indicating spatial proximity under overexpression conditions. To assess endogenous colocalization during infection, mouse primary macrophages (PMs) infected with RSV A2 (MOI = 0.1) were immunostained for ARCN1 and IKKε, revealing clear signal overlap (Fig 4e). Given that ARCN1 is a coat protein complex I (COPI) subunit that recognizes dilysine retrieval motifs, reversibly associates with non–clathrin-coated Golgi vesicles, and is required for Golgi membrane budding, anterograde transport of biosynthetic cargo from the endoplasmic reticulum (ER) through the Golgi to the trans-Golgi network, as well as COPI-mediated retrograde Golgi-to-ER retrieval of dilysine-tagged proteins [38–40], we focused subsequent localization analyses on the ER–Golgi axis. In HeLa cells infected with RSV A2 (MOI = 0.1), compartmentalization was quantified by colocalization with ER and Golgi markers. Upon infection, ARCN1 exhibited pronounced ER colocalization and moderate Golgi colocalization, whereas IKKε was likewise enriched at the ER with comparatively weaker Golgi colocalization (Fig 4f–4g). Collectively, these findings indicate that the ARCN1–IKKε association is concentrated at the ER, with a contributory Golgi component during RSV infection. This ER-centric distribution supports a model in which the ER—and to a lesser extent the Golgi—serves as a platform for the interaction between ARCN1 and IKKε.

### ARCN1 negatively regulates the protein stability of IKKε

**Fig 5:**
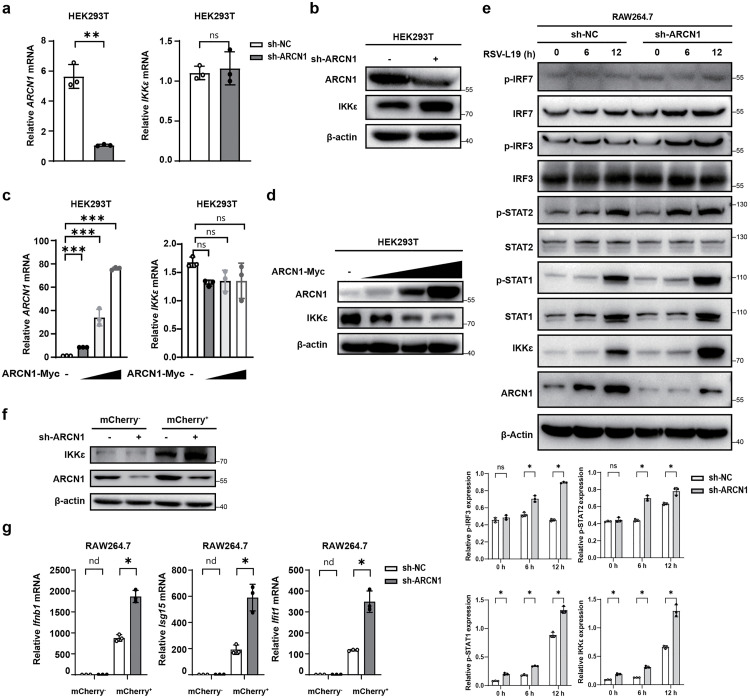
ARCN1 negatively regulates the protein stability of IKKε. **(a)** RT-qPCR was performed to detect the mRNA expressions of endogenous *ARCN1* and *IKK*ε in HEK293T cells transfected with sh-ARCN1 plasmids for 36 h. Data are presented as the mean ±SEM. n = 3 biological replicates. The expressions were normalized to 18S mRNA and further normalized to the sh-NC sample. **(b)** Western blot was used to detect the protein expressions of endogenous ARCN1 and IKKε in HEK293T cells transfected with sh-ARCN1 plasmids for 36 h. n = 3 biological replicates. **(c)** HEK293T cells were transfected with Co.Vec (ARCN1-Myc: -) or a gradient concentration of ARCN1-Myc plasmids for 36 h, and the mRNA expressions of *ARCN1* and *IKK*ε were detected by RT-qPCR. Data are presented as the mean ±SEM. n = 3 biological replicates. The expressions were normalized to 18S mRNA and further normalized to the Co.Vec sample. **(d)** HEK293T cells were transfected with Co.Vec (ARCN1-Myc: -) or a gradient concentration of ARCN1-Myc plasmids for 36 h, and the protein expressions of ARCN1 and IKKε were detected by Western blot. n = 3 biological replicates. **(e)** After RSV-L19 (MOI = 10) infected sh-NC-RAW264.7 and sh-ARCN1-RAW264.7 cells, the expressions of endogenous p-IRF7, IRF7, p-IRF3, IRF3, p-STAT2, STAT2, p-STAT1, STAT1, IKKε, and ARCN1 were detected by Western blot at different time points. n = 3 biological replicates. ImageJ software was used to perform quantitative grayscale analysis of Western blot bands, and the results were normalized to β-Actin protein bands. The statistical graphs were drawn. n = 3 biological replicates. **(f-g)** sh-NC-RAW264.7 and sh-ARCN1-RAW264.7 cells were infected with RSV-L19-mCherry (MOI = 10) for 24 h, followed by flow cytometric sorting to separate infected (mCherry⁺) and uninfected (mCherry⁻) cell populations. **(f)** The protein expression levels of ARCN1 and IKKε in different groups of cells were detected by Western blot analysis. n = 3 biological replicates. **(g)** The mRNA expression level of *Ifnb1, Isg15, and Ifit1* in different groups of cells was detected by RT-qPCR. Data are presented as means ± SEM. n = 3 biological replicates. Expression levels were normalized to 18S mRNA and further normalized to the sh-NC sample. (a, c)The *P*-value was determined using an unpaired t-test. (e, g) The *P*-value was determined using multiple unpaired t-tests. nd, not detected (below assay detection limit); ns, not significant (*P* > 0.05); *, *P* < 0.05; **, *P* < 0.01; ***, *P* < 0.001.

To elucidate the mechanism by which ARCN1 regulates IKKε, we verified the functional association between them via multidimensional experiments. RT-qPCR analysis revealed that *IKK*ε mRNA levels were unaffected by ARCN1 knockdown or overexpression in HEK293T cells (Fig 5a and 5c). In contrast, Western blot analysis revealed that ARCN1 knockdown significantly upregulated IKKε protein expression (Fig 5b), whereas gradient overexpression of ARCN1 dose-dependently downregulated IKKε protein expression (Fig 5d). Overall, these results indicate that ARCN1 downregulates IKKε protein stability.

To further explore ARCN1-mediated regulation of IKKε during RSV infection, sh-NC-RAW264.7 and sh-ARCN1-RAW264.7 cells were infected with RSV-L19 (MOI = 10) for 6 or 12 h. Western blot was used to assess the expression of IKKε and downstream signaling components, including phosphorylated and total IRF3, IRF7, STAT1, and STAT2. In addition, densitometric grayscale analysis of immunoblot bands was performed for IKKε, p-IRF3/7, and p-STAT1/2, and the results were normalized to β-Actin protein bands. Both Western blot and grayscale analysis showed that ARCN1 knockdown markedly increased IKKε protein abundance upon infection, accompanied by elevated phosphorylation of IRF3, STAT1, and STAT2 (Fig 5e).

Because fluorescence imaging indicated that, under these infection conditions (RSV-L19-mCherry, MOI = 10, 24 h), not all cells were infected (S1a Fig), we next sought to rigorously distinguish effects occurring in infected cells from potential bystander responses. Accordingly, RSV-L19-mCherry–infected cultures were subjected to flow cytometric sorting to isolate mCherry-positive (infected) and mCherry-negative (uninfected) populations from sh-NC-RAW264.7 and sh-ARCN1-RAW264.7 cells (S5a Fig). IKKε protein levels were assessed by Western blot, and *Ifnb1*, *Isg15*, and *Ifit1* mRNA levels were quantified by RT-qPCR. ARCN1 knockdown significantly increased IKKε protein abundance and upregulated *Ifnb1*, *Isg15*, and *Ifit1* expression exclusively in the infected population relative to sh-NC-RAW264.7 controls, with no significant changes in uninfected cells (Fig 5f–5g). These data indicate that the enhanced antiviral response induced by ARCN1 knockdown is confined to RSV-infected cells and is not mediated by bystander effects.

### ARCN1 induces IKKε degradation via the ubiquitin–proteasome pathway

**Fig 6:**
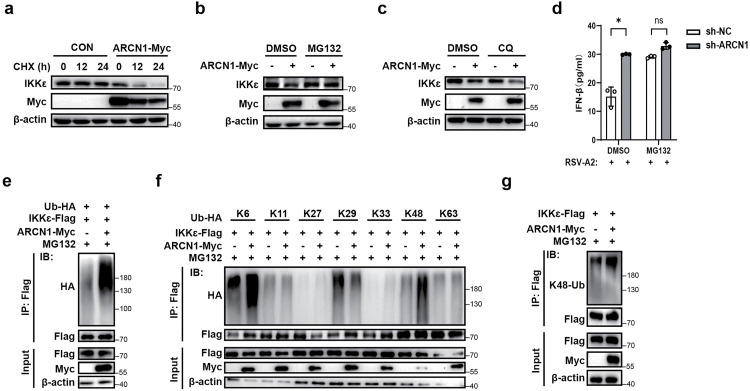
ARCN1 induces the degradation of IKKε via the ubiquitin-proteasome pathway. **(a)** HEK293T cells transfected with ARCN1-Myc plasmid for 36 h were treated with CHX (50 μM) at different time points, and the protein expressions of exogenous ARCN1 and endogenous IKKε were detected by Western blot. n = 3 biological replicates. **(b–c)** HEK293T cells transfected with ARCN1-Myc plasmid for 36 h were treated with proteasome inhibitor MG132 (10 μM) **(b)** or lysosome inhibitor CQ (20 μM) **(c)** for 12 h, and the protein expressions of exogenous ARCN1 and endogenous IKKε were detected by Western blot. n = 3 biological replicates. **(d)** ELISA of IFN-β secretion in supernatants from sh-NC- RAW264.7 and sh-ARCN1-RAW264.7 cells infected with RSV-A2 (MOI = 0.1) for 24 h and concomitantly treated with MG132 (10 μM) or control vehicle (DMSO). n = 3 biological replicates. **(e)** HEK293T cells were transfected with ARCN1-Myc, IKKε-Flag, and Ub-HA plasmids for 36 h, and the ubiquitination of exogenous IKKε was detected by Co-IP. All ubiquitination cell samples were treated with MG132 (10 μM) for 12 h before harvesting. n = 3 biological replicates. **(f)** The ubiquitination of exogenous IKKε was detected by Co-IP in HEK293T cells co-transfected with Ub-R6K-HA, Ub-R11K-HA, Ub-R27K-HA, Ub-R29K-HA, Ub-R33K-HA, Ub-R48K-HA, or Ub-R63K-HA along with ARCN1-Myc and IKKε-Flag for 36 h. All ubiquitination cell samples were treated with MG132 (10 μM) for 12 h before harvesting. n = 3 biological replicates. **(g)** HEK293T cells were transfected with ARCN1-Myc and IKKε-Flag for 36 h, and the K48-linked polyubiquitination of exogenous IKKε was detected by Co-IP. All ubiquitination cell samples were treated with MG132 (10 μM) for 12 h before harvesting. n = 3 biological replicates. **(d)** The *P*-value was determined using multiple unpaired t-tests. ns, not significant (*P* > 0.05); *P < 0.05.

To evaluate the impact of ARCN1 on IKKε protein stability, we performed cycloheximide (CHX) chase assays. Inhibition of the intracellular protein synthesis process revealed that ARCN1 overexpression significantly decreased IKKε protein levels (Fig 6a), indicating that ARCN1 promotes IKKε degradation. In eukaryotes, protein degradation occurs primarily via the ubiquitin–proteasome system, which targets specific intracellular ubiquitinated proteins, or the lysosome system, which degrades cytoplasmic components through autophagy [41].

Therefore, to identify the pathway responsible for ARCN1-mediated IKKε degradation, we treated ARCN1-overexpressing HEK293T cells with the proteasome inhibitor MG132 or the lysosomal inhibitor chloroquine (CQ). Western blot revealed that MG132, but not CQ, markedly blocked ARCN1-induced IKKε degradation (Fig 6b, 6c), indicating that ARCN1 promotes proteasome-dependent degradation of IKKε.

To further verify whether ARCN1 promotes IKKε degradation via the proteasomal pathway and thereby affects IFN-β secretion, ELISA was performed. sh-NC-RAW264.7 and sh-ARCN1-RAW264.7 cells were infected with RSV-A2 (MOI = 0.1) for 24 h and concomitantly treated with MG132 or control vehicle (DMSO); cell supernatants were collected to quantify secreted IFN-β. The results showed that under DMSO conditions, the IFN-β secretion of ARCN1 knockdown cells was significantly higher than that of control cells. However, after MG132 treatment, there was no significant difference in the IFN-β secretion levels between ARCN1 knockdown group and the control group (Fig 6d), suggesting that ARCN1 regulates IFN-β secretion by promoting the proteasomal degradation of IKKε. These data indicate that ARCN1 suppresses RSV-induced IFN-β secretion via proteasome-mediated degradation of IKKε, thereby negatively regulating downstream antiviral signaling.

As proteasome-dependent protein degradation is typically preceded by ubiquitination [42,43], we examined whether ARCN1 regulates IKKε ubiquitination. Western blot revealed that ARCN1 overexpression markedly enhanced polyubiquitination of exogenously expressed IKKε (IKKε-Flag) (Fig 6e). Further investigation into the specific type of polyubiquitin linkage indicated that ARCN1 predominantly promoted K48-linked polyubiquitination of IKKε-Flag, whereas other ubiquitin linkages remained unchanged (Fig 6f). Immunoblotting with a K48-specific ubiquitin antibody confirmed that ARCN1 significantly increased K48-linked polyubiquitination of IKKε-Flag (Fig 6g). Because K48-linked polyubiquitination usually leads to proteasome-mediated degradation [44,45], these results indicate that ARCN1 can induce K48-linked polyubiquitination and subsequent proteasomal degradation of IKKε. Taken together, these findings demonstrate that ARCN1 promotes K48-linked polyubiquitination of IKKε and its proteasomal degradation, thereby destabilizing IKKε and attenuating IFN-β production, ultimately dampening antiviral signaling during RSV infection.

### ARCN1 induces K48-linked polyubiquitination and proteasomal degradation of IKKε via STUB1

**Fig 7:**
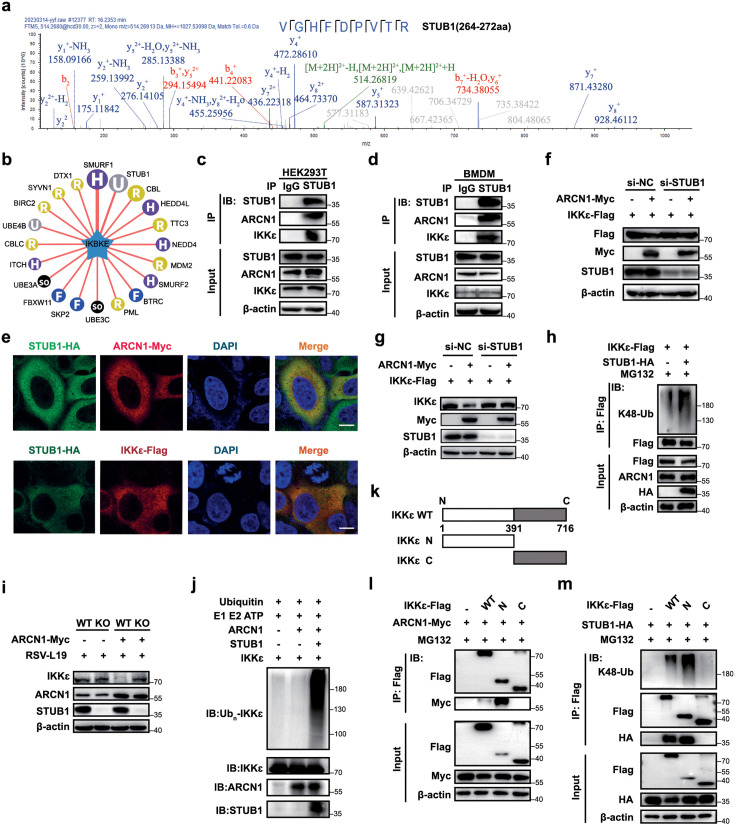
ARCN1 induces K48-linked polyubiquitination and proteasomal degradation of IKKε via STUB1. **(a)** HEK293T cells were transfected with ARCN1-Myc plasmid for 36 h, and the potential interactions between exogenous ARCN1 and endogenous E3 ubiquitin ligases were analyzed by Co-IP and mass spectrometry. The cell samples were treated with MG132 (10 μM) for 12 h before harvesting. The image displayed representative STUB1 peptides identified through mass spectrometry analysis, with *VGHFDPVTR* being one of the identified STUB1 peptides. **(b)** Ubibrowser E3 was used to predict the E3 ubiquitin ligases responsible for ubiquitinating IKKε (IKBKE). (http://ubibrowser.bio-it.cn/ubibrowser/). **(c–d)** Co-IP analysis of the interaction among endogenous STUB1, ARCN1, and IKKε in RAW264.7 **(c)** and BMDMs **(d)** was performed with an anti-STUB1 antibody. n = 3 biological replicates. **(e)** HeLa cells were co-transfected with STUB1-HA and either ARCN1-Myc or IKKε-Flag plasmids, followed by immunofluorescence staining using anti-HA (green) and anti-Myc (red) or anti-Flag (red) antibodies. Co-localization was analyzed by laser scanning confocal microscopy. Scale bar: 5 μm. (f) HEK293T cells were transfected with control or STUB1-siRNA for 24 h, followed by co-transfection with IKKε-Flag and ARCN1-Myc expression plasmids. After 48 h of the second transfection, the expressions of exogenous ARCN1 and IKKε protein expressions were analyzed by Western blot. n = 3 biological replicates. **(g)** HEK293T cells were transfected with control or STUB1-siRNA for 24 h, followed by co-transfection with IKKε-Flag and ARCN1-Myc expression plasmids for 48 h. The expressions of IKKε and exogenous ARCN1 protein were then analyzed by Western blot. n = 3 biological replicates. **(h)** HEK293T cells were transfected with STUB1-HA and IKKε-Flag for 36 h, and the K48-linked polyubiquitination of exogenous IKKε was detected by Co-IP. All ubiquitination cell samples were treated with MG132 (10 μM) for 12 h before harvesting. n = 3 biological replicates. **(i)** Wild-type (WT) and STUB1-knockout (KO) RAW264.7 cells were transfected with ARCN1-Myc and infected with RSV-L19 (MOI = 10) for 12 h. The endogenous IKKε protein levels were analyzed by Western blot. n = 3 biological replicates. **(j)** *In vitro* ubiquitination assays were performed with the indicated combinations of IKKε, ARCN1, and STUB1 (IKKε alone; IKKε + ARCN1; IKKε + ARCN1 + STUB1). Ubiquitinated IKKε was detected by immunoblotting using anti-ubiquitin and anti-IKKε antibodies. n = 3 biological replicates. **(k)** The diagram of IKKε protein domains. **(l)** HEK293T cells were co-transfected with ARCN1-Myc and IKKε-WT-Flag/IKKε-N-Flag/IKKε-C-Flag for 36 h, and the interaction was detected by Co-IP. n = 3 biological replicates. **(m)** HEK293T cells were co-transfected with STUB1-HA and IKKε-WT-Flag/IKKε-N-Flag/IKKε-C-Flag for 36 h, and the K48-linked polyubiquitination of exogenous IKKε-WT, IKKε-N, and IKKε-C was detected by Co-IP. The interaction between exogenous STUB1 and IKKε-WT/IKKε-N/IKKε-C was detected by Co-IP. n = 3 biological replicates.

The above results prompted an investigation into how ARCN1 regulates K48-linked polyubiquitination and subsequent proteasomal degradation of IKKε. Because ARCN1 lacks E3 ubiquitin ligase activity, we hypothesized that an E3 ubiquitin ligase bridges ARCN1 to IKKε. To identify the candidate ligase, Myc-tagged ARCN1 was immunoprecipitated from HEK293T cells and analyzed by mass spectrometry. These candidate E3 ligases were cross-referenced with predicted E3 ubiquitin ligases that target IKKε. The results identified the E3 ligase STUB1 (C terminus of Hsp70-interacting protein, CHIP), an E3 ubiquitin ligase known to mediate K48-linked polyubiquitination, as the main candidate [46,47] (Fig 7a, 7b). To verify the reliability of the mass spectrometry results, we performed endogenous protein interaction experiments in mouse bone marrow-derived monocytes (BMDMs) and HEK293T cells. The results showed that STUB1 interacts with both ARCN1 and IKKε (Fig 7c, 7d). Confocal microscopy results after immunofluorescence staining further showed cytoplasmic co-localization of all three proteins (Fig 7e).

To determine whether STUB1 is indispensable for ARCN1-mediated IKKε degradation, STUB1 was knocked down in HEK293T cells co-expressing IKKε-Flag and ARCN1-Myc. STUB1 depletion markedly attenuated the ARCN1-induced reduction of both exogenous IKKε-Flag and total IKKε protein levels (Fig 7f–7g), indicating that ARCN1 downregulates IKKε in a STUB1-dependent manner. Consistently, STUB1 overexpression markedly enhanced the K48-linked polyubiquitination of IKKε-Flag (Fig 7h). To more definitively demonstrate the importance of STUB1 in ARCN1-mediated IKKε degradation, STUB1-knockout (KO) RAW264.7 cells were generated. In STUB1-knockout (KO) RAW264.7 cells, ARCN1 overexpression failed to reduce endogenous IKKε levels following 12 h of RSV-L19 infection (MOI = 10), confirming that STUB1 is essential for ARCN1-mediated IKKε degradation (Fig 7i). Furthermore, we performed a reconstituted *in vitro* ubiquitination assay to assess ARCN1-dependent ubiquitination of IKKε. We compared three reaction conditions: no ARCN1-Myc added, ARCN1-Myc present alone, and ARCN1-Myc together with STUB1-HA, and analyzed ubiquitination of IKKε-Flag by immunoblotting. IKKε ubiquitination was undetectable in the absence of ARCN1 or with ARCN1 alone, and was observed only when both ARCN1 and STUB1 were present (Fig 7j), indicating that ARCN1-mediated ubiquitination of IKKε requires STUB1. Together, these results provide strong evidence that ARCN1 induces STUB1-dependent ubiquitination and degradation of IKKε. To further delineate the interactions among the three proteins, we constructed plasmids encoding the IKKε N-terminal kinase domain (IKKε-N, 1–390 aa), responsible for IRF3 phosphorylation and activation, and the C-terminal non-enzymatic domain (IKKε-C, 391–716 aa) (https://www.uniprot.org/uniprotkb/Q14164/entry) (Fig 7k). Co-IP and Western blot results showed that IKKε-N mediates the interaction with ARCN1 and STUB1 (Fig 7l, 7m). Simultaneously, we evaluated the extent of K48-linked polyubiquitination modification of each IKKε domain after STUB1 overexpression. The results showed that the lysine residues targeted by STUB1 for IKKε ubiquitination modification were located in IKKε-N (Fig 7m).

### STUB1 mediates K48-linked polyubiquitination of IKKε at K231 in an ARCN1-dependent manner

**Fig 8:**
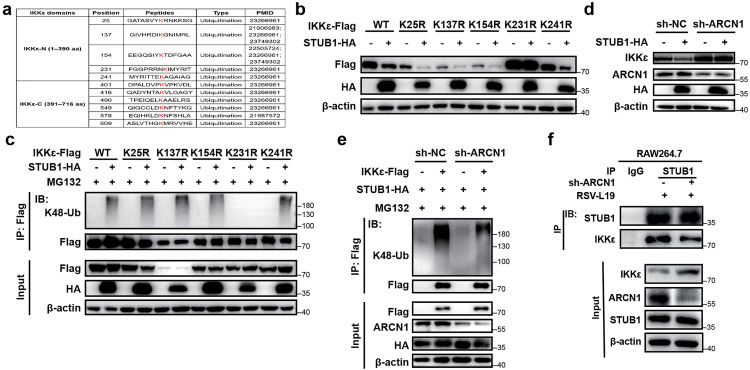
STUB1 mediates K48-linked polyubiquitination of IKKε at K231 in an ARCN1-dependent manner. **(a)** Predicted ubiquitination sites in the homo sapiens IKKε-N domain (1-390 aa). **(b)** HEK293T cells were transfected with IKKε-WT-Flag, IKKε-K25R-Flag, IKKε-K137R-Flag, IKKε-K154R-Flag, IKKε-K231R-Flag, and IKKε-K241R-Flag along with STUB1-HA for 36 h, and the protein expressions of exogenous STUB1 and IKKε were detected by Western blot. n = 3 biological replicates. **(c)** HEK293T cells were transfected with IKKε-WT-Flag, IKKε-K25R-Flag, IKKε-K137R-Flag, IKKε-K154R-Flag, IKKε-K231R-Flag, and IKKε-K241R-Flag, along with STUB1-HA for 36 h, and the K48-linked polyubiquitination of exogenous IKKε was detected by Co-IP. All ubiquitination cell samples were treated with MG132 (10 μM) for 12 h before harvesting. n = 3 biological replicates. **(d)** HEK293T cells were co-transfected with the control plasmid or ARCN1-shRNA plasmid along with STUB1-HA for 36 h, and the protein expressions of endogenous IKKε and ARCN1 were detected by Western blot. n = 3 biological replicates. **(e)** HEK293T cells were co-transfected with a control plasmid or ARCN1-shRNA plasmid along with STUB1-HA and IKKε-Flag for 36 h, and the K48-linked polyubiquitination of exogenous IKKε was detected by Co-IP. All ubiquitination cell samples were treated with MG132 (10 μM) for 12 h before harvesting. n = 3 biological replicates. **(f)** Co-immunoprecipitation of endogenous STUB1 and IKKε was performed in sh-NC-RAW264.7 and sh-ARCN1-RAW264.7 cells transfected with control shRNA or shRNA-targeting ARCN1 (sh-ARCN1) for 36 h, followed by RSV-L19 (MOI = 10) infection for 12 h using an anti-STUB1 antibody. n = 3 biological replicates.

We next aimed to identify the specific lysine sites for STUB1-induced IKKε ubiquitination. Given that STUB1 interacted with the IKKε-N domain and promoted its ubiquitination (Fig 7m), we initially mutated five lysine residues in the IKKε-N domain (Fig 8a) based on the predicted human IKKε ubiquitination modification sites (http://plmd.biocuckoo.org/view.php). IKKε-Flag variant plasmids carrying point mutations K25R, K137R, K154R, K231R, and K241R were constructed. Our results showed that STUB1 could still significantly downregulate the protein levels of four IKKε mutants, similar to wild-type IKKε (Fig 8b). However, the IKKε-K231R mutation significantly attenuated STUB1-induced IKKε protein degradation (Fig 8b), suggesting that STUB1 promotes K48-linked ubiquitination of IKKε at the K231 site. Consistent with this result, the K231R mutation significantly inhibited STUB1-mediated K48-linked ubiquitination of IKKε (Fig 8c). Together, these results suggest that STUB1 promotes K48-linked ubiquitination of IKKε at the K231 site, thereby mediating IKKε protein degradation.

To further determine whether STUB1-mediated ubiquitination of IKKε requires ARCN1, we first knocked down ARCN1 in HEK293T cells and then overexpressed STUB1. Western blot revealed that ARCN1 knockdown significantly attenuated STUB1-induced endogenous IKKε protein degradation (Fig 8d) and K48-linked polyubiquitination modification of IKKε (Fig 8e). Consistently, Co-IP analysis showed that the interaction between endogenous STUB1 and IKKε was markedly reduced in ARCN1-knockdown RAW264.7 cells following 12 h of RSV-L19 infection (MOI = 10) (Fig 8f). This further supports that ARCN1 acts as a critical adaptor that facilitates STUB1-mediated ubiquitination and degradation of IKKε during RSV infection. Collectively, these findings indicate that STUB1 mediates the ubiquitination and degradation of IKKε in an ARCN1-dependent manner.

### ARCN1 mediates the interaction between IKKε and STUB1

**Fig 9:**
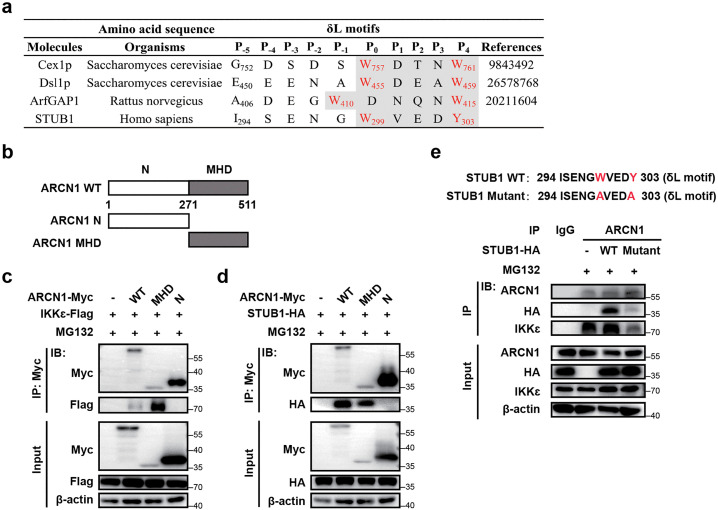
ARCN1 mediates the interaction between IKKε and STUB1. **(a)** The δL motifs (indicated in gray) contained in molecules from different organisms. The aromatic amino acid W/Y/F residues directly involved in binding ARCN1 were highlighted in red. **(b)** The diagram of ARCN1 protein domains. **(c)** HEK293T cells were co-transfected with IKKε-Flag and ARCN1-WT-Myc/ARCN1-MHD-Myc/ARCN1-N-Myc for 36 h, and the interaction was detected by Co-IP. All cell samples were treated with MG132 (10 μM) for 12 h before harvesting. n = 3 biological replicates. **(d)** HEK293T cells were co-transfected with STUB1-HA and ARCN1-WT-Myc/ARCN1-MHD-Myc/ARCN1-N-Myc for 36 h, and the interaction was detected by Co-IP. All cell samples were treated with MG132 (10 μM) for 12 h before harvesting. n = 3 biological replicates. **(e)** Co-IP analysis of the interaction between ARCN1 and exogenous STUB1-WT/STUB1-mutant was performed with anti-ARCN1 antibody after HEK293T cells were transfected with STUB1-WT-HA or STUB1-mutant-HA for 36 h. All cell samples were treated with MG132 (10 μM) for 12 h before harvesting. n = 3 biological replicates.

Previous results demonstrated that STUB1, IKKε, and ARCN1 interact in the cytoplasm and STUB1 mediates IKKε ubiquitination and degradation in an ARCN1-dependent manner. Therefore, we sought to investigate the underlying mechanism by which ARCN1 coordinates the interaction between STUB1 and IKKε. ARCN1, a subunit of the COPI complex, participates in intra-Golgi trafficking and retrograde transport from the Golgi apparatus to the ER. It recognizes and anchors target proteins to the outer membrane of the ER through its MHD domain [48,49]. Notably, although STUB1 lacks a fixed organelle localization, previous research has shown that it predominantly localizes to the outer membrane of the ER [50]. This observation raises the possibility that ARCN1 facilitates the recruitment of STUB1 to the ER membrane. The MHD domain of ARCN1 can recognize and interact with the YXXΦ motif [51,52] and WXn (1–6) [Y/W/F] motif (also known as the δL motif) in target proteins [48,49,53,54]. The δL motif is present in the C-terminal tail of STUB1 as well as in three other previously reported molecules [48,49,53] (Fig 9a).

Based on these findings, we speculated that ARCN1 recognizes the δL motif of STUB1 through its MDH domain, thereby directly interacting with STUB1. To verify this hypothesis, we constructed plasmids encoding the N-terminal domain of ARCN1 (ARCN1-N; 1–270 aa) and the ARCN1-MDH domain (271–511 aa), both tagged with Myc (https://www.uniprot.org/uniprotkb/P48444/entry#structure) (Fig 9b). Consistent with our hypothesis, Co-IP and Western blot revealed that ARCN1-MDH mediates its interaction with IKKε and STUB1 (Fig 9c, 9d).

Mutating the two aromatic amino acid residues (W/Y/F) in the δL motif of target proteins to alanine (A) can significantly reduce the binding of ARCN1 to the target proteins [48]. To further validate our hypothesis, we performed site-directed mutagenesis to construct a STUB1 mutant plasmid (STUB1-Mutant-HA; W299A and Y303A) in which the δL motif of STUB1 was altered. Immunoprecipitation and Western blot revealed that mutation of the δL motif significantly weakened the interaction among ARCN1, STUB1, and IKKε (Fig 9e). In summary, these findings demonstrate that the interaction between the δL motif of STUB1 and the ARCN1-MDH domain is fundamental to the interaction between ARCN1, STUB1, and IKKε.

### ARCN1 scaffolds the IKKε-STUB1 complex to suppress IFN-β signaling during RSV infection

**Fig 10:**
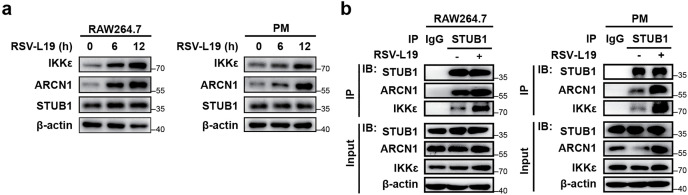
ARCN1 scaffolds the IKKε-STUB1 complex to suppress IFN-β signaling during RSV infection. **(a)** The protein expressions of IKKε, ARCN1, and STUB1 were detected by Western blot after RAW264.7 and PMs were infected with RSV-L19 (MOI = 10) at different time points. n = 3 biological replicates. **(b)** Co-IP analysis of the interaction between STUB1 and ARCN1/IKKε was performed using an anti-STUB1 antibody after RAW264.7 and PMs were infected with RSV-L19 (MOI = 10) for 12 h. n = 3 biological replicates.

Based on the above findings, we next addressed two key questions: (1) Does RSV-induced upregulation of ARCN1 affect the protein expression of STUB1? and (2) Does RSV infection alter the interaction among STUB1, ARCN1, and IKKε? To investigate this, RAW264.7 and mouse PMs were infected with RSV-L19 (MOI = 10), followed by Western blot to assess the expression of ARCN1, IKKε, and STUB1 with or without RSV infection. The results showed that RSV-L19 infection significantly increased ARCN1 and IKKε protein levels, whereas STUB1 expression remained unchanged (Fig 10a). Similar results were observed in RAW264.7 and mouse PMs infected with RSV-A2 (MOI = 0.1) (S6a Fig), indicating that RSV-induced ARCN1 upregulation does not affect STUB1 protein levels.

Furthermore, Co-IP and Western blot revealed a markedly enhanced interaction between ARCN1, STUB1, and IKKε in both RAW264.7 cells and PMs following RSV-L19 infection (MOI = 10) compared with uninfected controls (Fig 10b). These findings indicate that RSV infection strengthens the ARCN1–STUB1 interaction, which promotes STUB1-mediated ubiquitination of IKKε. This post-translational modification triggers accelerated proteasomal degradation of IKKε, consequently suppressing IFN-β signaling and ultimately increasing RSV viral load.

## Discussion

IFN-Is, especially IFN-β, are the first-line innate immune defense against RSV [30,34,55,56]. They play a key role in inhibiting viral replication and activating downstream antiviral immune responses. Therefore, understanding the regulatory mechanisms governing IFN-β production during RSV infection is essential, as it may reveal novel immune strategies against RSV and potential therapeutic targets. In this study, we demonstrated that RSV-induced upregulation of ARCN1 depends on IFN-β production and signaling through its receptor IFNAR.

**Fig 11:**
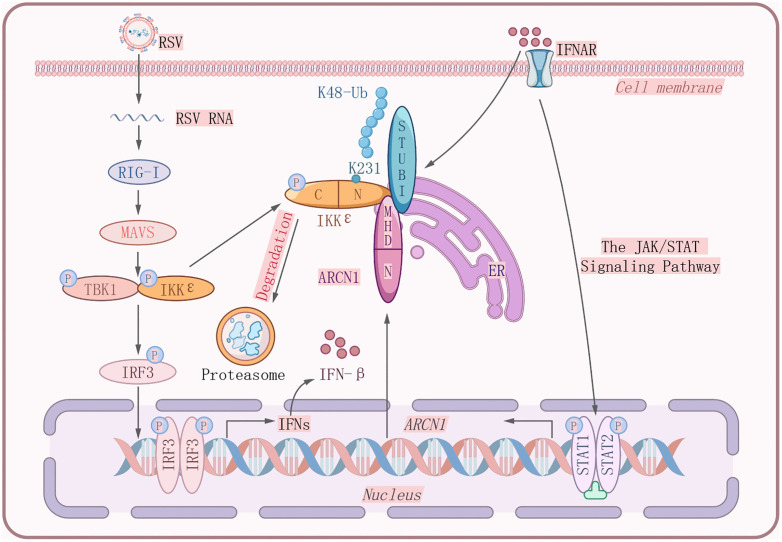
ARCN1 promotes STUB1-mediated IKKε degradation to suppress IFN-β signaling during RSV infection.

To our knowledge, our findings demonstrate for the first time that ARCN1 functions as a negative regulator of IFN-β production during RSV infection by facilitating the IKKε degradation. Mechanistically, ARCN1 expression was upregulated in response to IFN-β signaling during RSV infection. The upregulated ARCN1 interacts with the E3 ubiquitin ligase STUB1 at the ER through its MHD domain, which recognizes the δL motif of STUB1. Acting as an adaptor, ARCN1 promotes STUB1-mediated K48-linked polyubiquitination of IKKε at K231, leading to its proteasomal degradation. Given that IKKε is a key kinase downstream of RIG-I signaling, its degradation leads to impaired IRF3 phosphorylation and reduced IFN-β production.Importantly, the upregulation of ARCN1 following RSV infection likely represents a host-driven self-protective mechanism. Excessive or prolonged IFN-I signaling can amplify host pulmonary inflammation and tissue injury during RSV infection [25,26]. Thus, the induction of ARCN1 may restrain overactivation of the IFN-I pathway and prevent the associated immunopathology. Thus, IFN-β not only induces ARCN1 expression but is also negatively regulated by ARCN1 in a feedback manner, fine-tuning the antiviral response. This regulatory circuit ultimately attenuates IFN-β signaling and maintains host immune homeostasis (Fig 11).

As the COPI subunit responsible for recognizing targets, ARCN1 bridges STUB1 and IKKε through its flexible MHD domain, which recognizes the δL motif of STUB1 and binds to IKKε via an unknown mechanism. Our findings complement previous work by Annemarie et al. [24], who reported that COPA deficiency enhances IFN-I production by aberrantly activating cGAS-STING signaling due to impaired STING retrieval from the Golgi to the ER. Although that study also reported that ARCN1 and COPE deficiencies similarly potentiate cGAS-STING–induced IFN-I responses, it did not investigate the RNA sensor–mediated the IFN-I pathway. In contrast, our results reveal that ARCN1 knockdown in RAW264.7 cells enhanced IFN-β production upon stimulation with RSV, VSV, or the synthetic RNA mimic poly(I:C), suggesting that ARCN1 regulates IFN-β production through RNA sensor–mediated signaling pathways.

Furthermore, we demonstrated that ARCN1 exerted its negative regulatory effect by facilitating STUB1-mediated K48-linked polyubiquitination and subsequent proteasomal degradation of IKKε. Given that IKKε is a critical effector downstream of multiple pattern recognition receptors (PRRs), including RNA sensors such as MDA5, RIG-I, and TLR3, as well as the DNA sensor cGAS-STING [57], our findings suggest that ARCN1 plays a broader and fundamental role in modulating IFN-β production than previously recognized.

Previous studies have demonstrated that STUB1 functions as a negative regulator of antiviral immunity by mediating the ubiquitination and degradation of RIG-I [46,58–60], MAVS [61], and IRF-1 [62]. Consequently, either its interaction with target proteins or its expression is tightly regulated during viral infection. Given that ARCN1 lacks intrinsic E3 ubiquitin ligase activity, we hypothesized that an E3 ligase mediates the interaction between ARCN1 and IKKε. Our results supported this hypothesis, showing that ARCN1 overexpression significantly reduced IKKε protein levels without altering its mRNA expression, suggesting a post-translational regulatory mechanism. Considering STUB1’s established role in protein ubiquitination, the observation that ARCN1, STUB1, and IKKε interact with each other, and IKKε is predicted to be a substrate of STUB1, we identified STUB1 as the candidate E3 ligase. Our data confirmed that STUB1 mediates K48-linked polyubiquitination and subsequent IKKε degradation during RSV infection. Notably, STUB1 protein levels remained unchanged after RSV infection, but its interaction with IKKε was significantly enhanced upon ARCN1 upregulation, which further promoted STUB1-mediated K48-linked ubiquitination of IKKε. These findings confirm a previously unrecognized negative role for STUB1 in anti-RSV immunity and provide new mechanistic insights into how ARCN1 facilitates IKKε degradation.

The principal function of ARCN1 is to mediate intra-Golgi trafficking and protein transport from the trans-Golgi network to the ER. ARCN1 recognizes and anchors target proteins to the outer ER membrane via its flexible MHD domain [40,49,63]. Herein, we demonstrated that ARCN1 also performs an adaptor-like role by bridging STUB1 and IKKε. The ARCN1-MHD domain recognizes and interacts with the YXXΦ motif [51,52] and WXn(1–6)[Y/W/F] motif (known as the δL motif) [48,49,53,54] in target proteins. Furthermore, our experiments demonstrated that STUB1 interacts with ARCN1 via its δL motif. Studies have shown that STUB1 can ubiquitinate ER-resident proteins such as CYP3A4 [64] and CYP2E1 [65] and participates in ER-associated degradation and the unfolded protein response by mediating the ubiquitination and degradation of misfolded proteins [66–68] and inositol-requiring enzyme 1 (IRE1) [50], one of the three key sensors that reside on the ER membrane.

Although the essential role of ARCN1 in intracellular protein transport leads to embryonic lethality upon homozygous deletion [38,69], which limits the establishment of ARCN1 knockout cell lines and mouse models, we successfully generated stable ARCN1 knockdown cell lines and performed transient transfections to modulate its expression. These approaches provided substantial evidence that ARCN1 negatively regulates host interferon production and signaling through the ARCN1–STUB1–IKKε axis, offering new mechanistic insights and potential directions for the future translational study of ARCN1.

Given that many known STUB1 targets are localized at or around the endoplasmic reticulum (ER) membrane, we speculated that STUB1 may be recruited to the ER membrane or retrieved from the Golgi apparatus to exert its function. ARCN1 may facilitate this process by recognizing the δL motif within STUB1 through its MHD domain. This interaction enables ARCN1 to coordinate the positioning of STUB1 for the targeted degradation of substrates such as IKKε, thereby acting as a negative regulator of innate antiviral signaling pathways. Our observation of colocalization of endogenous ARCN1 and IKKε with the ER and the Golgi during RSV infection also supports this hypothesis. Specifically, the E3 ubiquitin ligase STUB1 mediates K48-linked polyubiquitination of IKKε, marking it for proteasomal degradation. During RSV infection, upregulated ARCN1 enhances its interaction with STUB1 via the MHD domain, potentially aiding the intracellular transport and ER location of STUB1. This further facilitates STUB1-induced K48-linked polyubiquitination and proteasomal degradation of IKKε.

Given these mechanistic insights, we further explored the role of ARCN1 in diverse immune contexts and its potential clinical significance. Our data demonstrate that ARCN1 overexpression suppresses IFN-β signaling in macrophages by promoting STUB1-mediated degradation of IKKε, thereby creating a favorable environment for RSV replication. This inhibitory effect is particularly pronounced in immune cells with robust interferon responses, such as macrophages, whereas in airway epithelial HEp-2 cells—characterized by inherently weak interferon responses—the impact of ARCN1 is relatively limited. In immunocompromised patients, elderly individuals, or preterm infants, where baseline interferon levels are already low, the immunosuppressive action of ARCN1 may further diminish antiviral capacity, facilitating RSV replication and exacerbating disease severity. Based on these findings, therapeutic strategies could aim to suppress ARCN1 activity or selectively inhibit STUB1 to restore IFN-I signaling without disrupting ARCN1’s essential roles in vesicular trafficking and cellular homeostasis. Direct use of IKKε agonists may also be another viable strategy. To achieve precise delivery and minimize systemic toxicity, future approaches could employ inhalable nanoparticle formulations or immune-cell-targeted delivery systems to administer ARCN1/STUB1 inhibitors or IKKε activators directly to airway macrophages. Such interventions could be integrated with existing anti-RSV therapies, including mRNA-based vaccines and adjuvanted protein subunit formulations [3,4], to yield synergistic effects by boosting host immunity, suppressing viral replication, and improving clinical outcomes. Notably, there are currently no commercially available inhibitors for ARCN1/STUB1 or agonists for IKKε, highlighting a pressing need for drug discovery efforts in this area and providing a clear direction for future research.

In summary, our study identifies ARCN1 as a critical host factor that triggers RSV infection by bridging STUB1 and IKKε to promote IKKε degradation and suppress IFN-β production. These findings reveal a novel mechanism of ARCN1-mediated suppression of innate immunity and suggest that targeting the ARCN1–STUB1–IKKε axis may represent a promising therapeutic strategy to enhance host antiviral immunity.

## Materials and methods

### Ethics statement

This study involving human participants and samples was reviewed and approved by the Ethics Committee of the Children’s Hospital Affiliated to Soochow University (Approval Number: 2022CS108). All research was performed in accordance with the Declaration of Helsinki. The experimental protocols were reviewed and approved by the Animal Ethics Committee of Soochow University (Approval No. SYXK2015–0018).

### Mice

All experimental mice were of the C57BL/6J background and purchased from the Experimental Animal Center of Soochow University. All mice were bred and maintained under specific pathogen-free (SPF) conditions at the Experimental Animal Center of Soochow University. All animal experiments were conducted in strict accordance with the Guide for the Care and Use of Laboratory Animals published by the National Institutes of Health [70].

### Reagents and antibodies

Recombinant mouse IFN-β (mIFN-β; R&D Systems) and polyinosinic-polycytidylic acid(Poly(I:C), Merck, P9582) were used at a concentration of 1 μg/mL. MG132 (MedChemExpress, HY-13259C) was used at a concentration of 10 μM. Chloroquine (CQ; MedChemExpress, HY-17589A) was used at a concentration of 20 μM. The antibodies used in the study were as follows: ARCN1 (ABclonal, A14993), ARCN1 (Santa, sc-514104), STUB1 (ABclonal, A11751), IRF3 (Cell Signaling Technology, D83B9), IKKε (CST, 3416S), IKKε (Santa, sc-376114), BAP31 (Proteintech, 11200–1-AP), Giantin (Abcam, ab37266), VSV-G tag (Abcam, ab1874), β-actin (Affinity, T0022), HA tag (Affinity, T0050), Myc tag (Affinity, AF6054), Flag tag (Affinity, T0003). Anti-respiratory syncytial virus M2-1 antibody (Abcam, ab94805) and anti-respiratory syncytial virus NS1 antibody (Invitrogen, MA5–50511). The secondary antibodies used were as follows: Alexa Fluor 647-conjugated anti-mouse IgG (Invitrogen, A21235) and Alexa Fluor 488-conjugated anti-rabbit IgG (SouthernBiotech, 4050-30).

### Plasmids

Human ARCN1-Myc, ARCN1-N-Myc, and ARCN1-MHD-Myc were purchased from Changsha Ruiying Biotechnology Co., Ltd. The shARCN1-mouse/human plasmids were constructed via in-frame insertion of the shARCN1-targeting sequences into the lentivirus encoding pLL3.7 vector. The shARCN1-targeting sequences are available in S2 Table. Human IKKε-Flag, IKKε-N-Flag, and IKKε-C-Flag were purchased from Changsha Ruiying Biotechnology Co., Ltd. IKKε-K25R-Flag, IKKε-K137R-Flag, IKKε-K154R-Flag, IKKε-K231R-Flag, and IKKε-K241R-Flag (The lysine site mutant plasmids for IKKε) were constructed through site-directed mutagenesis. Plasmids expressing the IFN-I pathway upstream molecules (tagged with Flag) were kindly provided by Dr. Chunsheng Dong (Soochow University). Ubiquitin-HA (Ub-HA), Ub-R6K-HA, Ub-R11K-HA, Ub-R27K-HA, Ub-R29K-HA, Ub-R33K-HA, Ub-R48K-HA, and Ub-R63K-HA (all lysines on the ubiquitin gene are mutated to arginines except the corresponding lysine) were kindly provided by Dr. Hui Zheng (Soochow University). The IFN-β promoter reporter plasmid and Renilla plasmid were kindly provided by Dr. Jianfeng Dai (Soochow University). Human STUB1-HA and STUB1-Mutant-HA (W299A and Y303A) were purchased from Changsha Ruiying Biotechnology Co., Ltd.

### Cells and viruses

RAW264.7, HEK293T, HeLa, Vero, and HEp-2 cells were purchased from the American Type Culture Collection (ATCC). ARCN1 knockdown (sh-ARCN1 RAW264.7) and control RAW264.7 (sh-NC RAW264.7) stable cell lines were constructed by using the lentiviral packaging technology. All cell cultures were maintained in a humidified atmosphere at 37°C under 5% CO_2_ atmosphere and cultured in Dulbecco’s modified Eagle’s medium (DMEM; HyClone) supplemented with 10% fetal bovine serum (FBS; Biological Industries), 100 U/mL penicillin (NCM Biotech), and 100 μg/mL streptomycin (NCM Biotech). Sendai virus (SeV) and VSV were kindly provided by Dr. Jianfeng Dai (Soochow University). Respiratory syncytial virus strain L19 (RSV-L19-mCherry) was kindly provided by Dr. Chunsheng Dong (Soochow University). Respiratory syncytial virus strain A2 (ATCC, VR-1540) was also used in the study.

### Isolation of mouse macrophages

Bone marrow-derived macrophages (BMDMs) were isolated from the bone marrow of 6–8-week-old wild-type C57BL/6J mice. The bone marrow cells were collected from the femurs and tibias of the experimental mice, and BMDMs were differentiated in RPMI-1640 medium with 10% FBS, including 20 ng/mL mouse macrophage colony-stimulating factor (M-CSF; Pepro Tech, Rocky Hill, NJ) for 7 days. Primary PMs were isolated from 6- to 8-week-old wild-type C57BL/6J mice 4 days after receiving an intraperitoneal injection of 4% thioglycollate (BD) and then subsequently cultured in RPMI 1640 medium supplemented with 10% FBS [30].

### RNA isolation and RT‐qPCR assay

Total RNAs were extracted from cells using TRIzol reagent (Vazyme) as per the manufacturer’s instructions. All cDNA was synthesized from 1 μg of total RNA by using the HiScriptIII RT SuperMix for RT-qPCR (Vazyme, R323-01). Then, the mRNA levels were analyzed by RT‐qPCR with specific gene primers using RT-qPCR SYBR Green Master Mix (Yeasen, 11203ES). The primer sequences are listed in S3 Table.

### Mass spectrometry

RAW264.7 cells were either infected or left uninfected with RSV-L19 (MOI = 10) and cultured for 24 h. The cells were lysed in a buffer containing 150 mM NaCl, 10 mM HEPES, 8 M urea, and 1% PMSF (Sigma). Lysis was enhanced by two rounds of sonication on an ice bath using a high-intensity ultrasonic processor (Scientz), followed by continued incubation on an ice bath for 30 min. The lysates were centrifuged at 12,000 × *g* for 15 min at 4°C, and the supernatant was collected and transferred into 2 mL of mass spectrometry-grade centrifuge tubes. The protein concentrations were determined by using a BCA protein assay kit (Thermo Fisher). For reduction, 2 mM dithiothreitol (DTT; 0.6 μL) was added to the protein solution, followed by incubation at 37°C for 45 min. Alkylation was conducted by adding 50 mM chloroacetamide (CAA; 2.4 μL) and incubating at room temperature in the dark for 45 min. The protein solution was then diluted with HEPES buffer to a final protein concentration of 4 μg/μL and a final urea concentration of <2 M. A total of 200 μg protein was then digested with 4 μg trypsin (Thermo Fisher) at 37°C for 24 h.

After digestion, 15 μg of the peptide mixture was adjusted to the same final volume with HEPES buffer and acidified by adding an equal volume of 0.1% trifluoroacetic acid (TFA). The peptides were then desalted and enriched using C18 ZipTips. The final peptide samples were analyzed by LC-MS/MS using a Dionex liquid chromatography system coupled with an Orbitrap Elite mass spectrometer (Thermo Fisher). MS/MS spectra were processed using Proteome Discoverer software (v1.2) and searched against the UniProt database paired with a reverse decoy database. The fragment ion mass tolerance was set at 0.02 Da. The identified proteins were subjected to further bioinformatics analyses, which included functional annotation, classification, and enrichment analysis.

Mass spectrometry data were deposited in the iProx repository (http://www.iprox.cn) under the accession number IPX0013952000.

### Immunofluorescence and confocal microscopy analyses

HeLa cells were transfected with plasmids for 48 h before analysis by immunofluorescence microscopy. The images were acquired at 100 × magnification. Briefly, the cells were fixed at room temperature with 4% paraformaldehyde, followed by permeabilization with 0.5% Triton X-100 and blocking with 3% bovine serum albumin (BSA). The treated cells were then incubated overnight at 4°C with primary antibodies as per the experimental requirements; these antibodies included anti-Myc (Affinity, AF6054), anti-Flag (Affinity, T0003), and anti-HA (Affinity, T0050). After washing, the cells were incubated with secondary antibodies, including Alexa Fluor 488-conjugated goat anti-rabbit IgG (Southern Biotech, 4050-30) and Alexa Fluor 647-conjugated goat anti-mouse IgG (Invitrogen, A21235). The nuclei were counterstained with DAPI. Fluorescence images were captured with a Nikon confocal microscope.

### Luciferase reporter assay

HEK293T cells were co-transfected with IFN-β promoter reporter plasmid, Renilla control plasmid, the IFN-I pathway molecule expression plasmids (e.g., RIG-I-N, MAVS, TBK1), and ARCN1-Myc plasmid or empty vector control for 48 h. After transfection, the medium was replaced with fresh DMEM supplemented with 10% FBS. The luciferase activity was determined by using the Dual-Luciferase Reporter Assay System (Vazyme, DD1205-01) in accordance with the manufacturer’s instructions. The firefly luciferase activity was normalized to the Renilla luciferase activity to calculate the transfection efficiency.

### Western blot and Co-IP

First, the cells were lysed on an ice bath for 30 min using NP-40 lysis buffer (Beyotime) supplemented with 1 mM phenylmethylsulfonyl fluoride (PMSF; Beyotime). For Co-IP, whole-cell extracts were collected 48 h after transfection and lysed under the same conditions. The lysates were centrifuged at 12,000 × *g* for 20 min at 4°C, and the resulting supernatants were incubated overnight at 4°C with magnetic beads conjugated to anti-Flag, anti-Myc, or anti-HA antibodies (Bimake), or with Protein G agarose beads (Roche). The beads were then washed twice with NP-40 wash buffer and once with high-salt wash buffer. The bound proteins were eluted by boiling the beads in the 5 × SDS loading buffer (NCM Biotech) for 10 min. For Western blot, the protein samples were separated by SDS-PAGE and transferred onto PVDF membranes. The membranes were blocked with 5% non-fat milk in PBST at room temperature for 1 h, followed by incubation with specific primary antibodies. Signal detection was performed through enhanced chemiluminescence (ECL; FDbio, FD8020).

### Children’s peripheral blood samples

PBMCs from children in the RSV-infected and control groups were isolated from residual clinical blood samples originally collected for routine diagnostic purposes by the Medical Laboratory Department of the Children’s Hospital of Soochow University. A total of 24 peripheral blood samples were collected from RSV-infected children with bronchiolitis hospitalized at the Children’s Hospital of Soochow University between 1 November 2021 and 31 December 2021. All samples were obtained from children aged 0–2 years. In addition, 24 age-matched Children with non-RSV-infected illnesses were used as the control group (S4 Table). The diagnosis and severity evaluation of bronchiolitis were performed in accordance with the Clinical Practice Guidelines for the Management of Bronchiolitis in Children (2021). RSV infection was confirmed via RSV antigen tests of nasopharyngeal aspirates. Furthermore, other microbiological tests were conducted to exclude other respiratory tract infections. No other pathogens were detected by these tests. Peripheral blood samples were obtained from the patients upon admission. The study was approved by the ethics committee of the Children’s Hospital of Soochow University. Written informed consent was obtained from at least one guardian of each patient before their enrollment. The data from patients were analysed anonymously.

### Flow cytometric sorting

Flow cytometric sorting was performed to separate RSV-infected and uninfected cell populations based on the intrinsic mCherry fluorescence encoded by RSV-L19-mCherry. shNC-RAW264.7 and shARCN1-RAW264.7 cells were infected with RSV-L19-mCherry (MOI = 10) for 24 h, harvested, and resuspended in phosphate-buffered saline (PBS) containing 2% FBS. Flow cytometric analysis and sorting were performed using the BD FACSAria II cell sorter (BD Biosciences, USA). The PerCP-Cy5.5 channel was used to detect the mCherry signal, enabling the identification and isolation of mCherry⁺ (RSV-infected) and mCherry⁻ (uninfected) populations. Uninfected RAW264.7 cells were used as negative controls for gating. The sorted cells were collected in complete medium and immediately processed for protein and RNA extraction for subsequent Western blot and qPCR analyses.

### Establishment of STUB1-knockout RAW264.7 cell line via electroporation-based CRISPR/Cas9 gene editing

Three CRISPR guide RNAs (gRNAs) targeting the mouse STUB1 were designed by using the Genscript online CRISPR design tool; their sequences are listed in S2 Table. The gRNAs were combined with GenCRISPR Cas9 v1.2 protein (Genscript) in electroporation buffer (Celetrix) and incubated at room temperature for 10 min to allow ribonucleoprotein (RNP) complex formation. The RAW264.7 cells were washed with 1 × PBS, centrifuged, and resuspended in the electroporation mixture containing the RNP complexes. The cell suspension was then transferred into a Celetrix electroporation cuvette and electroporated by using a Celetrix electroporator at 530 V. Following electroporation, the cells were immediately transferred into DMEM supplemented with 10% FBS (without penicillin and streptomycin) and cultured for 72 h. The successful knockout of the STUB1 was confirmed by Western blot in the RAW264.7 cell line.

### *In vitro* protein expression and ubiquitination assays

*In vitro* protein expression was performed by using the TNT T7 Quick Coupled Transcription/Translation System (Promega, USA) in accordance with the manufacturer’s instructions. Plasmids containing a T7 promoter, including ARCN1-Myc, IKKε-Flag, and STUB1-HA served as templates. Each reaction (50 μL total volume) contained 40 μL TNT-T7 Quick Master Mix, 1 μL 1 mM methionine, 2 μg Plasmid DNA Template, 1 μL T7 TNT PCR Enhancer, and Nuclease-Free Water to achieve a final volume of 50 μL. The mixture was gently mixed, briefly centrifuged, and incubated in a water bath at 30°C for 60–90 min. The protein expression was confirmed by Western blot, and the remaining reactions were stored at −80°C until further use.

*In vitro* ubiquitination assays were performed by using a Ubiquitination Kit (Enzo Life Sciences, USA) following the manufacturer’s protocol. Proteins expressed *in vitro* were used directly as substrates. The reaction mixture (50 μL total) contained 10 × buffer (5 μL), 10 × Ubiquitin (5 μL), 20 × E1 (2.5 μL), 10 × E2 (2.5 μL), STUB1-HA (10 μL), ARCN1-Myc (10 μL), IKKε-Flag (10 μL), Mg^2+^-ATP (2.5 μL), and nuclease-free water to volume. The mixture was gently vortexed, briefly centrifuged, and incubated in a water bath at 37°C for 6–8 h. The reactions were terminated by adding protein-loading buffer and heating at 70°C for 10 min. Ubiquitination of the target proteins was then assessed by Western blot.

### Statistical analysis

Statistical analyses were performed using an unpaired two-tailed *t*-test, multiple unpaired *t*-tests, or a two-way analysis of variance (ANOVA) test. Data are presented as the mean ±standard error of the mean (SEM) from three independent experiments. *P* < 0.05 was considered to indicate statistical significance. The significance levels are denoted as follows: nd, not detected (below assay detection limit); ns, not significant (*P > 0.05*); * P < 0.05; ** P < 0.01; *** P < 0.001; **** P < 0.0001.

## Supporting information

S1 Fig RSV-L19 successfully infects macrophages. (a) The fluorescence of RSV-L19 was detected by fluorescence microscopy after RAW264.7 and PMs were infected with RSV-L19-mCherry (MOI = 10) at different time points. Scale bar = 200 μm. ImageJ software was used to perform quantitative statistical analysis of the red fluorescence signal, and statistical graphs were drawn. n = 3 biological replicates. The *P*-value was determined using an unpaired t-test. ****, *P* < 0.0001.
(TIF)

S2 Fig RSV-L19 infection leads to increased ARCN1 expression in macrophages. (a–b) The mouse macrophage cell line RAW264.7 cells were infected with RSV-L19 (MOI = 10) for 24 h. The protein expression profiles of infected and uninfected RAW264.7 cells were analyzed by protein mass spectrometry. (a) Hierarchical clustering of differentially expressed proteins in RSV-L19-infected RAW264.7 cells. (b) The heatmap displaying the expression profiles of traffic proteins in RSV-L19-infected and control RAW264.7 cells. n = 3 biological replicates.
(TIF)

S3 Fig RSV-A2 infection leads to increased ARCN1 expression in macrophages. (a) The mRNA expression of *Ifnb1* and *RSV-F* were detected by RT-qPCR after RAW264.7 were infected with RSV-L19 (MOI = 10) or RSV-A2 (MOI = 0.1) for 12 h. Data are presented as means ± SEM. n = 3 biological replicates. Expression levels were normalized to 18S mRNA and further normalized to the PBS treatment. (b) The protein expression level of RSV M2-1 was detected by Western blot after RAW264.7 were infected with RSV-L19 (MOI = 10) or RSV-A2 (MOI = 0.1) for 12 h. n = 3 biological replicates. (c–d) The protein expression level of ARCN1 was detected by Western blot after RAW264.7 (c) and PMs (d) were infected with RSV-A2 (MOI = 0.1) at different time points. n = 3 biological replicates. (e–f)The mRNA expression levels of *Ifnb1* and *Arcn1* were detected by RT-qPCR after RAW264.7 (e) and PMs (f) were infected with RSV-A2 (MOI = 0.1) at different time points. Data are presented as means ± SEM. n = 3 biological replicates. Expression levels were normalized to 18S mRNA and further normalized to the 0-h time point. (a, e, f) The *P*-value was determined using an unpaired t-test. ns, not significant (*P* > 0.05); *, *P* < 0.05; **, *P* < 0.01; ***, *P* < 0.001.
(TIF)

S4 Fig ARCN1 is successfully overexpressed and knocked down in target cells. (a) The mRNA expression of *Arcn1* in sh-NC-RAW264.7 and sh-ARCN1-RAW264.7 was detected by RT-qPCR. Data are presented as the mean ±SEM. n = 3 biological replicates. The expressions were normalized to 18S mRNA and further normalized to the sh-NC sample. (b) The protein expression of ARCN1 in sh-NC-RAW264.7 and sh-ARCN1-RAW264.7 was detected by Western blot. n = 3 biological replicates. (c) HEK293T cells were transfected with control vector (Co.Vec) or ARCN1-Myc (ARCN1) plasmids for 36 h. The mRNA expression of *ARCN1* in the cells was detected by RT-qPCR. The data are presented as the mean ±SEM. n = 3 biological replicates. The expressions were normalized to 18S mRNA and further normalized to the Co.Vec sample. (d) HEK293T cells were transfected with control vector (Co.Vec) or ARCN1-Myc (ARCN1) plasmids for 36 h. The protein expression of ARCN1 in the cells was detected by Western blot. n = 3 biological replicates. (e) HEK293T cells were transfected with lentivirus encoding pLL3.7 vector (sh-NC) or shRNA plasmid specific for ARCN1 (sh-ARCN1) for 36 h. The mRNA expression of *ARCN1* in the cells was detected by RT-qPCR. Data are presented as the mean ±SEM. n = 3 biological replicates. The expressions were normalized to 18S mRNA and further normalized to the sh-NC sample. (f) HEK293T cells were transfected with lentivirus encoding pLL3.7 vector (sh-NC) or shRNA plasmid specific for ARCN1 (sh-ARCN1) for 36 h. The protein expression of ARCN1 in the cells was detected by Western blot. n = 3 biological replicates. (g) HEp-2 cells were transfected with control vector (Co.Vec) or ARCN1-Myc (ARCN1) plasmids for 36 h. The mRNA expression of *ARCN1* in the cells was detected by RT-qPCR. Data are presented as the mean ±SEM. n = 3 biological replicates. The expressions were normalized to 18S mRNA and further normalized to the Co.Vec sample. (h) HEp-2 cells were transfected with control vector (Co.Vec) or ARCN1-Myc (ARCN1) plasmids for 36 h. The protein expression of ARCN1 in the cells was detected by Western blot. n = 3 biological replicates. (a, c, e, g) The *P*-value was determined using an unpaired t-test. *P < 0.05; ****P < 0.0001.
(TIF)

S5 Fig Flow cytometric sorting of RSV–L19–infected RAW264.7 cells. (a) RSV-infected (mCherry⁺) cell populations were isolated from sh-NC-RAW264.7 and sh-ARCN1-RAW264.7 cells infected with RSV-L19-mCherry (MOI = 10) for 24 h. The PerCP-Cy5.5 channel was used to detect the mCherry signal. Uninfected cells were used as negative controls for gating. n = 3 biological replicates.
(TIF)

S6 Fig RSV-A2 infection increases ARCN1 and IKKε protein levels without affecting the STUB1 expression. (a) The protein expressions of IKKε, ARCN1, and STUB1 were detected by Western blot after RAW264.7 and PMs were infected with RSV-A2 (MOI = 0.1) at different time points. n = 3 biological replicates.
(TIF)

S1 Table RSV-A2 vs RSV-L19 comparison summary. (DOCX)

S2 Table The sh-ARCN1 targeting sequences and the gRNA sequence targeting STUB1. (DOCX)

S3 Table Sequences of primers used in RT-qPCR. (DOCX)

S4 Table Demographic of bronchiolitis patients and controls. (DOCX)
