# Supplementary material for: ARCN1 suppresses innate immune responses against respiratory syncytial virus by promoting STUB1-mediated IKKε degradation
Source: PLoS Pathog. 2025 Dec 4;21(12):e1013751. doi: 10.1371/journal.ppat.1013751 (PMC12677500; doi:10.1371/journal.ppat.1013751)
Supplement: S1 Table — (DOCX) [file ppat.1013751.s007.docx]

**Supplementary Table**

**S1 Table.** **RSV-A2 vs RSV-L19 comparison summary**

| **Feature** | **RSV-A2 (Wild-type)** | **RSV-L19 (RSV Line 19)** | **PMID** |
| --- | --- | --- | --- |
| **Origin** | Isolated 1961 (Melbourne, Australia); established as prototypic RSV-A strain for vaccine models. | Isolated 1967 (University of Michigan); may be a Long strain variant adapted through mouse passage. | **31117229; 19211758** |
| **Replication kinetics (in vitro)** | High replication in HEp-2, Vero, A549, and airway epithelial cultures; faster cytopathicity. | Replicates at lower titers in HEp-2 cells and mouse lungs than A2. | **19211758; 16936271** |
| **Thermal / structural stability** | Less stable in pre-fusion F configuration. | More stable F protein, possibly due to six unique amino acid substitutions. | **31117229; 19211758** |
| **Cytopathology / Histology** | Mild inflammation, limited goblet cell metaplasia in BALB/c mice. | Strong airway mucus, goblet cell hyperplasia, airway plugging. | **19211758; 16936271** |
| **Immune bias (TH1 vs. TH2)** | TH1-dominant (higher IFN-γ, IL-10); weaker IL-13 response. | TH2-skewed (high IL-13, mucus gene expression). | **19211758; 16936271** |
| **Type I IFN response** | Early, strong IFN-β induction. | Weaker IFN-β induction during early infection. | **19211758** |
| **Viral load (in vivo)** | Moderate lung viral titers; peaks early and declines rapidly. | Lower than A2; not correlated with disease severity. | **19211758; 16936271** |
| **Airway Hyperreactivity (AHR)** | No significant AHR observed. | Strong, dose-dependent AHR; abolished in IL-13⁻/⁻ mice. | **19211758; 16936271** |
| **Pathogenicity in mice** | Moderate disease, mainly TH1-type inflammation. | Severe disease, mucus overproduction, AHR. | **19211758; 16936271** |
| **Experimental Use** | Vaccine platform and structural biology standard. | Model for mucus-dominant RSV disease; used for fluorescent visualization(RSV-L19-mCherry). | **31117229** |
