# Supplementary material for: ARCN1 suppresses innate immune responses against respiratory syncytial virus by promoting STUB1-mediated IKKε degradation
Source: PLoS Pathog. 2025 Dec 4;21(12):e1013751. doi: 10.1371/journal.ppat.1013751 (PMC12677500; doi:10.1371/journal.ppat.1013751)
Supplement: S2 Table — (DOCX) [file ppat.1013751.s008.docx]

# Supplementary Table

**S2 Table.** **The sh-ARCN1 targeting sequences and the gRNA sequence targeting STUB1**

| **Name** | **Sequences (5’-3’)** |
| --- | --- |
| Murine sh-*Arcn1* forward | tgctgagatgcgtcgtaaagcattcaagagatgctttacgacgcatctcagcttttttc |
| Murine sh-*Arcn1* reverse | tcgagaaaaaagcggcgtaaagcaaaggaatttctcttgaaaattcctttgctttacgccgca |
| Human sh-ARCN1 forward | tgctgagatgcgtcgtaaagcattcaagagatgctttacgacgcatctcagcttttttc |
| Human sh-ARCN1 reverse | tcgagaaaaaagctgagatgcgtcgtaaagcatctcttgaatgctttacgacgcatctcagca |
| Murine STUB1 gRNA | cgtgggccgcaagtacccgg |
