# Supplementary material for: ARCN1 suppresses innate immune responses against respiratory syncytial virus by promoting STUB1-mediated IKKε degradation
Source: PLoS Pathog. 2025 Dec 4;21(12):e1013751. doi: 10.1371/journal.ppat.1013751 (PMC12677500; doi:10.1371/journal.ppat.1013751)
Supplement: S3 Table — (DOCX) [file ppat.1013751.s009.docx]

# Supplementary Table

**S3 Table. Sequences of primers used in RT-qPCR**

| **Gene** | **primer (5’-3’)** | **Reverse primer (5’-3’)** |
| --- | --- | --- |
| Human *β-actin* | cattgctgacaggatgcagaagg | tgctggaaggtggacagtgagg |
| Human *IFN-β* | cattacctgaaggccaagga | cagcatctgctggttgaaga |
| Murine *Ifnb1* | atgagtggtggttgcaggc | tgacctttcaaatgcagtagattca |
| Murine *β-actin* | cattgctgacaggatgcagaagg | tgctggaaggtggacagtgagg |
| Murine *Arcn1* | gccttcggaaagtggaaatggc | tcgatctcaccaatcacaggcg |
| Human *ARCN1* | ggagagtggaaatggctgtgatg | tcgatctcaccgataacaggcg |
| *18S* | cggctaccacatccaaggaa | gctggaattaccgcggct |
| Murine *Isg15* | ggtgtccgtgactaactccat | tggaaagggtaagaccgtcct |
| Human *ISG15* | gggacctgacggtgaagatg | cgccgatcttctgggtgat |
| Murine *Ifit1* | ctgagatgtcacttcacatggaa | gtgcatccccaatgggttct |
| Human *IFIT1* | cacaagccattttctttgct | acttggctgcatatcgaaag |
| *RSV-F* | gaattgcagttgctcatgcaa | tggcgattgcagatccaaca |
| Human *IKKɛ* | ggctacaacgaggagcagattc | ggacgcttgatacttctgcacg |
| Human *STUB1* | tcaaggagcagggcaatcgtct | gcatcttcaggtagcacaaggc |
