# Supplementary material for: ARCN1 suppresses innate immune responses against respiratory syncytial virus by promoting STUB1-mediated IKKε degradation
Source: PLoS Pathog. 2025 Dec 4;21(12):e1013751. doi: 10.1371/journal.ppat.1013751 (PMC12677500; doi:10.1371/journal.ppat.1013751)
Supplement: S4 Table — (DOCX) [file ppat.1013751.s010.docx]

# Supplementary Table

**S4 Table.** **Demographic of bronchiolitis patients and controls**

| **Variables** | **Controls** | **Bronchiolitis** |
| --- | --- | --- |
| No. of subjects | 24 | 24 |
| Age, yrs | 1.15 ±0.40 | 1.25 ±0.52 |
| Male, n (%) | 14 (58.33%) | 15 (62.5%) |
| Respiratory Pathogen Antibody | RSV-IgM Negative | RSV-IgM Positive |
